# Genome Reduction Is Associated with Bacterial Pathogenicity across Different Scales of Temporal and Ecological Divergence

**DOI:** 10.1093/molbev/msaa323

**Published:** 2020-12-12

**Authors:** Gemma G R Murray, Jane Charlesworth, Eric L Miller, Michael J Casey, Catrin T Lloyd, Marcelo Gottschalk, Alexander W (Dan) Tucker, John J Welch, Lucy A Weinert

**Affiliations:** 1 Department of Veterinary Medicine, University of Cambridge, Cambridge, United Kingdom; 2 Department of Genetics, University of Cambridge, Cambridge, United Kingdom; 3 European Bioinformatics Institute, Wellcome Genome Campus, Cambridge, United Kingdom; 4 Département de Pathologie et Microbiologie, Université de Montréal, Montréal, QC, Canada; 5 Warwick Medical School, University of Warwick, Coventry, United Kingdom; 6 Haverford College, Haverford, PA, USA; 7 School of Mathematical Sciences, University of Southampton, Southampton, United Kingdom

**Keywords:** reductive genome evolution, pathogenicity, bacterial evolution, endosymbionts

## Abstract

Emerging bacterial pathogens threaten global health and food security, and so it is important to ask whether these transitions to pathogenicity have any common features. We present a systematic study of the claim that pathogenicity is associated with genome reduction and gene loss. We compare broad-scale patterns across all bacteria, with detailed analyses of *Streptococcus suis*, an emerging zoonotic pathogen of pigs, which has undergone multiple transitions between disease and carriage forms. We find that pathogenicity is consistently associated with reduced genome size across three scales of divergence (between species within genera, and between and within genetic clusters of *S. suis*). Although genome reduction is also found in mutualist and commensal bacterial endosymbionts, genome reduction in pathogens cannot be solely attributed to the features of their ecology that they share with these species, that is, host restriction or intracellularity. Moreover, other typical correlates of genome reduction in endosymbionts (reduced metabolic capacity, reduced GC content, and the transient expansion of nonfunctional elements) are not consistently observed in pathogens. Together, our results indicate that genome reduction is a consistent correlate of pathogenicity in bacteria.

## Introduction

The emergence of new bacterial pathogens is a major threat to human health and food security across the globe (Vouga and Greub 2016). Although every instance of pathogen emergence will be unique in some way, identifying common features could help us to understand, predict, and ultimately prevent these transitions to pathogenicity. One intriguing observation is that some of the most serious human pathogens have smaller genomes and fewer genes than their closest nonpathogenic or less pathogenic relatives (Pupo et al. 2000; Ochman and Moran 2001; Moran 2002; Stinear et al. 2008; Toft and Andersson 2010; Georgiades and Raoult 2011; Langridge et al. 2015; Weinert and Welch 2017). Nevertheless, without formal comparative studies, it is difficult to know whether these are isolated instances of genome reduction, or part of a broader trend (Weinert and Welch 2017).

There are also doubts about whether genome reduction has anything to do with pathogenicity per se (Weinert and Welch 2017). Most notably, similar patterns of genome reduction are found in mutualist or commensal bacteria that have adopted a host-restricted or intracellular lifestyle. In these endosymbiotic bacteria, genome reduction appears as part of a process that often includes a decreased proportion of G/C relative to A/T bases, a preferential loss of genes in metabolic pathways, and a transient expansion in the proportion of the genome that is nonfunctional (because of pseudogenization or the proliferation of selfish elements). This “endosymbiont syndrome” is a plausible outcome of evolution in small isolated populations, where natural selection is less effective and opportunities for gene exchange and homologous recombination are reduced; coupled with a relaxation of some selection pressures, due to a dependency on the host (Mira et al. 2001; Moran 2002; Newton and Bordenstein 2011; McCutcheon and Moran 2012; Rao et al. 2015; Bobay and Ochman 2018). As such, it has been suggested that the genome reduction observed in pathogens may be a consequence of the intracellular or host-restricted lifestyle that they share with endosymbionts, rather than their pathogenicity (Moran 2002).

**Fig. 1.**
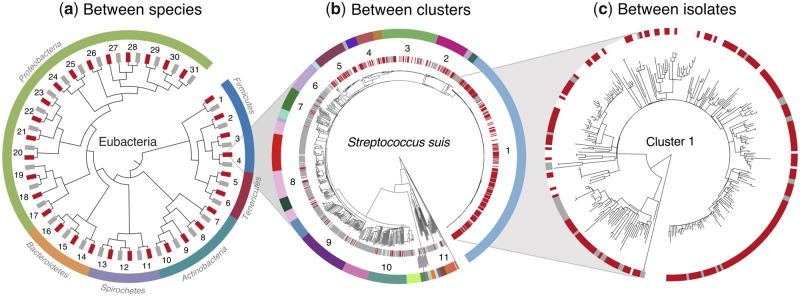
The evolution of pathogenicity over three evolutionary scales: (*a*) between species of bacteria, (*b*) between clusters of *Streptococcus suis*, and (*c*) between isolates of *S. suis* within clusters. (*a*) A cladogram of our 31 pairs of congeneric species, comprising a pathogen (red) and a nonpathogen (gray). Numbers refer to table 1 and suprageneric relationships are from (Zhu et al. 2019). (*b*) A core genome phylogeny of our 1,079 isolates of *S. suis*. Individual disease (red) and carriage (gray) isolates are indicated in the inner strip. The outer strip describes the 34 genetic clusters, with the 11 “mixed clusters” that include multiple disease and carriage isolates numbered. (*c*) An illustrative phylogeny of our largest and most pathogenic cluster, constructed from a recombination-stripped local core genome alignment. Individual disease (red) and carriage (gray) isolates are again indicated on the strip.

Here, we present a systematic study of genome reduction and pathogenicity in bacteria across multiple scales of ecological and temporal divergence (fig. 1). First, at the broadest scale, we compare pairs of bacterial species, where a known vertebrate pathogen has a nonpathogenic relative in the same genus. These data span six distinct phyla and include a wide range of pathogen ecologies with variable degrees of host restriction (fig. 1*a*, table 1). Second, we focus on *Streptococcus suis*, an opportunistic pathogen of pigs, whose pathogenicity has previously been associated with genome reduction (Weinert et al. 2015; Vötsch et al. 2018). This bacterium is of particular interest because it has undergone multiple independent transitions between carriage and disease forms, yet both forms are generally extracellular, and have equal levels of host restriction. As such, we can observe “replicated” changes in pathogenicity that are not accompanied by changes in the broader ecology (see below). Furthermore, the species includes multiple genetic clusters of closely related isolates that vary in their association with disease. By comparing patterns between clusters (fig. 1*b*), and between isolates within clusters (fig. 1*c*), we can contrast long- and short-term changes.

**Table 1.** Between-Species Data Set.

Phylum	Pair	Genus	Pathogen sp. (#)	Nonpathogen sp. (#)
Firmicutes	1	*Clostridium*	*tetani* (2)	*carboxidivorans* (1)
	2*	*Bacillus*	*anthracis* (31)	*subtilis* (39)
	3*	*Streptococcus*	*pneumoniae* (28)	*thermophilus* (8)
	4	*Streptococcus*	*suis* (19)	*oligofermentans* (1)
Tenericutes	5	*Mycoplasma*	*putrefaciens* (2)	*yeatsii* (1)
	6	*Mycoplasma*	*hyopneumoniae* (2)	*flocculare* (1)
Actinobacteria	7*	*Rhodococcus*	*equi* (1)	*pyridinivorans* (1)
	8^§^	*Corynebacterium*	*diphtheriae* (13)	*efficiens* (1)
	9*	*Mycobacterium*	*abscessus (28)*	*smegmatis* (7)
	10*^§^	*Mycobacterium*	*leprae* (1)	*indicus* (1)
Spirochaetes	11	*Treponema*	*pallidum* (2)	*primitia* (1)
	12*	*Leptospira*	*interrogans* (8)	*biflexa* (2)
	13	*Brachyspira*	*hyodysenteriae* (2)	*murdochii* (1)
Bacteroidetes	14	*Bacteroides*	*helcogenes* (1)	*vulgatus* (1)
	15	*Flavobacterium*	*branchiophilum* (1)	*johnsoniae* (1)
	16	*Flavobacterium*	*columnare* (4)	*indicum* (1)
Proteobacteria	17^§^	*Brucella*	*suis* (15)	*ceti* (1)
	18	*Rickettsia*	*prowazekii* (9)	*e. I. scapularis* (1)
	19	*Rickettsia*	*rickettsii* (9)	*peacockii* (1)
	20	*Neisseria*	*gonorrhoeae* (9)	*lactamica* (1)
	21	*Burkholderia*	*pseudomallei* (46)	*thailandensis* (11)
	22	*Taylorella*	*equigenitalis* (2)	*asinigenitalis* (1)
	23*^§^	*Bordetella*	*pertussis* (51)	*hinzi* (2)
	24^§^	*Francisella*	*noatunensis* (6)	*philomiragia* (7)
	25	*Legionella*	*pneumophila* (21)	*fallonii* (1)
	26	*Aeromonas*	*salmonicida* (1)	*media* (1)
	27^§^	*Actinobacillus*	*pleuropneumoniae* (4)	*succinogenes* (1)
	28	*Haemophilus*	*influenzae* (16)	*parainfluenzae* (1)
	29^§^	*Citrobacter*	*rodentium* (1)	*amalonaticus* (3)
	30	*Yersinia*	*enterocolitica* (9)	*rohdei* (1)
	31	*Yersinia*	*pestis* (32)	*similis* (1)

Note.—Pairs with a known difference in levels of intracellularity (*) and/or host-restriction (§) between pathogen and nonpathogen.

## Results and Discussion

### Data Sets

For our broadest-scale between-species data set (fig. 1*a*), we carried out a systematic search for phylogenetically independent species pairs, comprising a vertebrate pathogen, a congeneric nonpathogen, and an outgroup, with publicly available whole-genome data. Our choice of pairs followed a strict protocol to minimize the influence of subjective choice (see Materials and Methods for full details). Following this protocol, we obtained 31 species pairs (table 1, supplementary table S1, Supplementary Material online) represented by 478 ingroup genomes (supplementary table S2, Supplementary Material online). These pairs spanned all six of the bacterial phyla that contain known pathogen species. They include obligate (e.g., *Mycobacterium leprae*) and opportunistic pathogens (e.g., *Streptococcus pneumoniae*); pathogens with different primary hosts (e.g., *Legionella pneumophila*); and pathogens with several (e.g., *Yersinia pestis*), and no (e.g., *Taylorella equigenitalis*) close pathogenic relatives. These data therefore represent a breadth of pathogenic ecologies and evolutionary histories. Because the species pairs differed both in their sampling densities (i.e., the number of available genomes), and in the evolutionary distances between the species, we developed a phylogenetic comparative method to properly account for these differences.

For our within-species comparisons (fig. 1*b* and *c*), we collated 1,079 whole genomes of *S. suis*, collected across three continents (table 2, supplementary table S3, Supplementary Material online). Data were associated with clinical information, and include “carriage isolates” from the tonsils of pigs without *S. suis* associated disease, and “disease isolates” from the site of infection in pigs with *S. suis* associated disease (divided into respiratory or systemic infections). We also included zoonotic disease isolates from humans with systemic disease in Vietnam, which a previous study found to be indistinguishable from isolates associated with systemic disease in pigs (Weinert et al. 2015). A core genome alignment was used to build a consensus phylogeny; but as *S. suis* is highly recombining, we inferred genetic clusters using an approach that does not assume a single evolutionary history across the genome (Corander et al. 2008; Tonkin-Hill et al. 2018). We identified 34 clusters, of which 33 contained isolates that could be unambiguously categorized as carriage or disease (fig. 1*b*, supplementary fig. S1*a* and table S4, Supplementary Material online). These clusters have variable levels of genetic diversity and include some with recent origins; for example, a previous study dated the origin of our largest and most pathogenic cluster (cluster 1, fig. 1*c*) to the 1920s (Weinert et al. 2015). There were marked differences between clusters in the proportion of disease isolates, and this correlated with the presence of virulence genes and serotypes with known disease associations (supplementary fig. S2, Supplementary Material online). Allowing for comparisons at the finest scale, 11/34 were “mixed clusters,” containing both multiple carriage isolates and multiple disease isolates, and these are numbered in figure 1*b*.

**Table 2.** *Streptococcus suis* Data Set.

Origin	# Isolates: Total (SP, RP, C)	Genome Size Range (Mb)	Genetic Cluster(s)	Dates of Collection	Reference(s)
UK	440 (43, 52, 205)	1.95–2.56	1–11, 13–14, 16–18, 20, 22–25, 27–28, 30–33	2009–2015	Weinert et al. (2015); Wileman et al. (2019)
Canada	197 (36, 31, 56)	1.92–2.49	1–3, 5–18, 20, 22–26, 29–30	1983–2016	Hadjirin et al. (2020)
China	197 (0, 0, 197)	2.06–2.54	1, 3, 5–12, 18–22, 27–30, 32, 34	2013–2014	Zou et al. (2018)
Vietnam	190 (149, 0, 32)	1.97–2.19	1	2000–2010	Weinert et al. (2015)
USA	16 (3, 4, 0)	2.02–2.46	3, 5, 8–10, 26	2016	Hadjirin et al. (2020)
Spain	10 (7, 0, 0)	2.03–2.42	1, 4, 8, 22	2016	Hadjirin et al. (2020)
Reference Collection	29 (14, 2, 3)	1.98–2.30	1–4, 6, 8, 13–14, 18–19	—	NCBI GenBank, http://www.ncbi.nlm.nih.gov/genbank (last accessed 2016)

Note.—SP, systemic pathogen; RP, respiratory pathogen; C, carriage.

### Pathogenicity Is Associated with Genome Reduction at All Divergence Scales

**Fig. 2.**
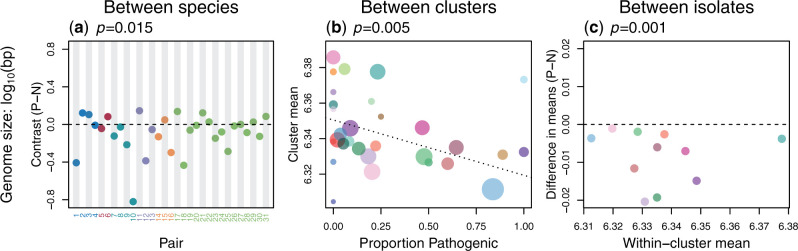
Pathogenicity is associated with smaller genomes. (*a*) Each point represents a phylogenetically independent species pair, with numbers and colors from figure 1*a*. Standardized contrast values are ordered such that negative values imply smaller genomes in the pathogenic species. *P*-value is from a permutation test of the null hypothesis of no difference in the mean contrast value (as indicated by the dotted line). (*b*) Each point represents a cluster of *Streptococcus suis* isolates, with colors from figure 1*b*, and sizes indicating the number of isolates. A sample-size weighted regression shows that clusters containing a larger proportion of pathogenic isolates have a smaller average genome size. (*c*) Each point shows the difference in mean genome size between disease and carriage isolates in a cluster of *S. suis* isolates, such that negative values imply smaller genomes in the pathogens. Each point corresponds to a “mixed cluster” (containing multiple disease and carriage isolates), with colors and numbers matching figure 1*b*. The *P*-value is from a permutation test as in (*a*).

Across all three scales of divergence, our data sets showed substantial variation in genome size, and in each case, we found a strong and negative association with pathogenicity. This is shown in figure 2. At the broadest scale, pathogenic species had smaller genomes than their nonpathogenic relatives more often than expected by chance (fig. 2*a*; see also supplementary table S5 and fig. S3*a*, Supplementary Material online for robustness analyses). Within *S. suis*, genetic clusters had smaller genomes when they contained a higher proportion of disease isolates (fig. 2*b*; see also supplementary table S6, Supplementary Material online). The same pattern held within clusters; in 11/11 mixed clusters, disease isolates had smaller genomes on average, than carriage isolates from the same cluster (fig. 2*c*).

Two further lines of evidence suggest that changing genome size is a cumulative process that persists for long periods of time. First, in our between-species data set, a Brownian motion model of genome size evolution provides a good fit, which demonstrates that more distantly related pairs have larger differences in genome size. This is shown in supplementary figure S4, Supplementary Material online. Second, in *S. suis*, between-cluster differences in genome size remain apparent when we consider the carriage isolates alone: carriage isolates have smaller genomes when they are found in clusters containing a higher proportion of disease isolates (“Data Set C” in supplementary table S6, Supplementary Material online). Finally, in *S. suis*, there is an association between genome size, and disease severity. We see the smallest genomes in isolates associated with more invasive systemic disease, with less severe respiratory disease isolates tending to have intermediate genome size. This is shown in supplementary figure S5, Supplementary Material online.

All of these results apply to total genome size. However, we observed the same pattern when we used genome annotations to consider only known functional elements. Across all divergence scales, pathogenicity was associated with fewer genes, and smaller coding length, as well as smaller genome size (supplementary fig. S3*a*–*i*, Supplementary Material online). In *S. suis*, a large fraction of genome reduction is due to the loss of mobile genetic elements (see supplementary figs. S7*a* and S8*a*–*c*, Supplementary Material online). However, this cannot account for all genome size variation, as genome reduction is also observed in the remainder of the genome, with mobile elements excluded (supplementary fig. S8*b* and *c*, Supplementary Material online).

### Pathogenicity Is Not Consistently Associated with an Endosymbiont Syndrome

In endosymbionts, genome reduction is frequently associated with a preferential loss of metabolic genes, a transient proliferation of nonfunctional DNA, and a reduction in GC content. Below, we ask whether these signatures also accompany the genome reduction observed in pathogens.

#### Metabolic Genes

**Fig. 3.**
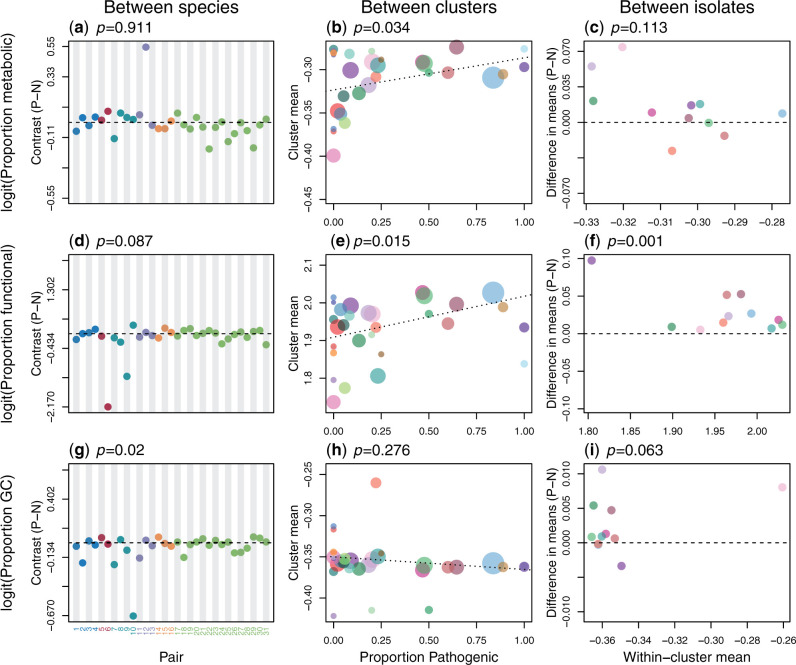
Pathogenicity is not consistently associated with the “endosymbiont syndrome”. Each panel tests for an association between pathogenicity and another signature of the endosymbiont syndrome, (*a*–*c*): the proportion of protein-coding genes with metabolic function, (*d*–*f*): the proportion of the genome with known function, (*g*–*i*): the proportion of the genome comprising GC base pairs. Negative contrasts (*a*, *c*, *d*, *f*, *g*, *i*) or negative regression slopes (*b*, *e*, *h*) are consistent with an endosymbiont syndrome. All other details match figure 2.

Metabolic genes are often lost in obligate endosymbionts, because they can instead rely on the host for certain nutrients, therefore reducing selective constraint on their own genes. This is especially true of intracellular symbionts that live in the nutrient-rich host cytoplasm (Moran 2002; Zientz, Dandekar et al. 2004). Although this process is expected to be especially prominent in mutualists, which might be actively provisioned by the host, pathogens might also acquire products from their hosts. However, we found no evidence to support this hypothesis. Having identified genes with metabolic function using COG categorization (see Materials and Methods), we found that pathogens did contain fewer metabolic genes over all three timescales (supplementary fig. S3*m*–*o* and table S5, Supplementary Material online). Nevertheless, as shown in figure 3*a*–*c*, these trends were no greater than would be expected, given the overall pattern of gene loss that we demonstrated above. In particular, between species, the proportion of genes with a metabolic function evolved gradually over time (supplementary fig. S6*j* and *k*, Supplementary Material online), but does not differ significantly between pathogenic and nonpathogenic congeners (fig. 3*a*, supplementary table S5, Supplementary Material online); whereas within *S. suis*, the proportion of metabolic genes is actually higher in pathogens, both between and within clusters (fig. 3*b* and *c*), suggesting that they are lost less rapidly than randomly chosen genes. This is the exact opposite of the predicted pattern. These analyses considered all metabolic genes as a single category. However, finer grained analyses, using individual COG categories associated with metabolism, also revealed no consistent pattern of preferential gene loss (supplementary table S7, Supplementary Material online). Between-cluster analysis in *S. suis* did show preferential loss of genes in two of the eight categories: energy production and conversion, and amino acid transport and metabolism. But neither result would survive correction for multiple testing; and for both categories, trends in the opposite direction were found within clusters of *S. suis*, and between species (supplementary table S7, Supplementary Material online). Together, results suggest that preferential loss of genes with a metabolic function is not a consistent correlate of pathogenicity. In fact, as shown in supplementary figure S9, Supplementary Material online, genome reduction in *S. suis* does not appear to involve the loss of any consistent set of genes. The most striking pattern is that putative virulence genes are more consistently present in pathogens, bucking the overall trend for gene loss (Weinert et al. 2015).

#### Nonfunctional Elements

Although very old endosymbionts are often gene dense (e.g., Nakabachi et al. 2006; McCutcheon and Moran 2007; Kuo et al. 2009), more recently established endosymbionts often show increased numbers of pseudogenes and/or an expansion of nonfunctional selfish elements. This results in a lower proportion of their genome being functional, suggesting a reduction in either the strength or efficacy of negative selection. To test whether this pattern also held in pathogens, we estimated the proportion of each of our genomes that contained functional sequence (see Materials and Methods). Results, shown in figure 3*d*–*f*, show no evidence of the predicted pattern on any of our three timescales. Between species, we found no consistent difference in the proportion between pathogens and nonpathogens (fig. 3*d*, supplementary table S5, Supplementary Material online). The evolution of this proportion also failed to fit a Brownian motion model of evolution (supplementary fig. S6*d* and *e*, Supplementary Material online), confirming that the accumulation of nonfunctional DNA is a transient process. Results in *S. suis* were again the opposite of the prediction. The proportion of the genome that was functional was consistently higher in pathogens (fig. 3*e* and *f*). As such, the within-species transitions to pathogenicity differed from the more recent transitions to symbiosis.

#### GC Content

The genomes of endosymbiotic bacteria often have lower GC contents than those of their free-living counterparts (Moran 2002). This could reflect metabolic adaptation to the host environment (Rocha and Danchin 2002; Dietel 2019), or a reduction in GC-biased gene conversion due to lower rates of recombination (Lassalle et al. 2015). But it is usually assumed to be a consequence of a reduced efficacy of selection, coupled with a bias towards GC-to-AT mutations (Hershberg and Petrov 2010; Hildebrand et al. 2010; McCutcheon and Moran 2012). Results shown in figure 3*g*–*i*, show that patterns of GC content in pathogens are more complicated than predicted. Between species, we did observe the predicted tendency for pathogens to be GC-poor (fig. 3*g*). However, further analysis showed that this result was attributable to a subset of pairs that had undergone a large-scale shift in ecology. In 12/31 pairs, the pathogenic species showed a greater degree of host restriction and/or intracellularity than its nonpathogenic congener (table 1), and the relationship between pathogenicity and GC held only for these pairs, and not for the remainder of the data (supplementary table S5, Supplementary Material online). Results for genome size showed the exact opposite pattern: the association between genome reduction and pathogenicity was driven by 19/31 pairs with similar ecologies (supplementary table S5, Supplementary Material online). (Results for metabolic genes and nonfunctional DNA showed no differences between either division of the data: supplementary table S5, Supplementary Material online.) As such, between species, the AT-richness of some pathogens might be explained by aspects of their ecology that they share with endosymbionts, but their genome reduction cannot be explained in this way.

Within *S. suis*, the raw results for GC showed no clear patterns (fig. 3*h* and *i*, supplementary fig. S1*c*, Supplementary Material online). However, this is attributable to divergent groups of *S. suis* with unusual GC content (Baig et al. 2015; see supplementary figs. S1*a*, *c*, and S7*b*, Supplementary Material online for details). Once these groups are removed, results suggest a balance of opposing forces relating to the mobile accessory genome, and to the core genome. We find that mobile genetic elements tend to have lower GC content (supplementary fig. S8*d*, Supplementary Material online). As such, their absence from isolates with smaller genomes tends to increase genome-wide GC (supplementary fig. S7*c*, Supplementary Material online). By contrast, the core genome shows the reverse pattern, with isolates with smaller genomes being less GC rich (supplementary fig. S7*d*, Supplementary Material online). But this is only evident between genetic clusters, suggesting that accumulation of GC-to-AT mutations in the core genome is a slow process. The result is that the predicted association between pathogenicity and low GC is observed in *S. suis*, but only between clusters, and only in the core genome. This is shown in supplementary figure S10, Supplementary Material online.

The decreased GC content in more pathogenic clusters of *S. suis* might be caused by reduction in the efficacy of selection, or fewer opportunities for GC-biased gene conversion. Consistent with these hypotheses, we find that more pathogenic cluster of *S. suis* have shorter terminal branches (supplementary fig. S11, Supplementary Material online), as well as evidence of faster rates of protein evolution, and lower rates of homologous recombination (supplementary fig. S12, Supplementary Material online; see also Weinert et al. 2015). We note that these changes cannot be due to increased host restriction or intracellularity, such as we have inferred in the between-species data. Another possibility is that they stem from bottlenecks associated with transmission between hosts (Kono et al. 2016; Sheppard et al. 2018). There is evidence of high rates of transmission in pathogenic *S. suis* in the broad geographic spread of the more pathogenic clusters, despite their relatively recent origin. For example, cluster 1 includes isolates from China, Vietnam, the UK, Spain and Canada (supplementary table S4, Supplementary Material online).

## Conclusion

We have demonstrated a statistical association between pathogenicity and genome reduction in bacteria. This association applies across bacterial phyla, across a wide range of pathogenic ecologies, and across different scales of divergence. We have also demonstrated that genome reduction in pathogens can occur without the correlates that are often observed in bacterial endosymbionts. In particular, we find no evidence of the preferential loss of genes with metabolic functions, which is predicted if genome reduction is driven by increased dependence on, or exploitation of, the host. We also find little evidence of maladaptive genome evolution involving the accumulation of nonfunctional elements. In fact, both trends go in the opposite direction in *S. suis*. We do find that genome reduction is sometimes associated with a reduction in GC content, but this signature is patchy. In the between-species data, it is only present when the pathogenic species has become more host-restricted or intracellular, and in *S. suis*, it is observed only over longer time periods and only in the core genome; perhaps due to bottlenecks associated with increased transmission. Genome reduction in bacterial pathogens is therefore distinct from that in endosymbionts, and cannot be solely attributed to features, such as host restriction or intracellularity. Consequently, genome reduction could prove a useful marker of emerging and increasing pathogenicity in bacteria.

## Materials and Methods

### Data Sets

For the between-species data set, we aimed for consistency, and so chose our data from a single common source: the NCBI RefSeq database (O’Leary et al. 2016; release 76, apart from one *Rickettsia peacockii* genome, added from release 77). We began by identifying all eubacterial genera that were represented by multiple named species in RefSeq. We then used Bergey (Whitman 2015), and the wider literature, to classify all species in these genera as “pathogens”, “nonpathogens”, or “ambiguous/unknown”. Pathogenicity was defined with respect to vertebrates, not only because vertebrate pathogenicity is better studied, but because vertebrate adaptive immunity is implicated in some theories of genome reduction (Weinert and Welch 2017). We note that all such designations must contain an element of uncertainty and ambiguity, not least because of the ubiquity of opportunistic pathogenicity. For this reason, we restricted our definition of “pathogens” to species that have repeatedly been reported to cause disease in immuno-competent vertebrate hosts, and preferred species where there was evidence of long-term persistence as a pathogen. For example, we scored *Staphylococcus aureus* as “ambiguous,” because human infection commonly occurs from carriage forms (Huang et al. 2011), and because carriage status could not be inferred from metadata associated with the sequenced isolates. Similarly, “nonpathogens” were defined as species that were known to be free-living or commensals, even if there were isolated cases of secondary infections. For example, *Aeromonas media* was designated as a nonpathogen, despite a single reported case of isolation, together with pathogenic *Yersinia enterocolitica* in a patient recovering from infection with *Aeromonas caviae* (Rautelin et al. 1995).

After these assignments, we aimed to choose pairs without further subjectivity, or influence of prior knowledge. As such, we used the following process. First, for each genus containing at least one pathogen and one nonpathogen, we downloaded all available complete genomes (see supplementary table S2, Supplementary Material online). (Draft genomes were excluded because we observed that they varied substantially in length for a few species.) We then used *Phylosift* (Darling et al. 2014) to align 37 single copy orthologs identified as universal to all bacteria. Concatenated alignments of these loci were checked and corrected by eye (available on Dryad at https://doi.org/10.5061/dryad.nzs7h44qc), and we used *MEGA7* (Kumar et al. 2016) to build neighbor-joining phylogenies using variation at synonymous sites. We used a modified Nei–Gojobori method using Jukes–Cantor and complete deletion of sites with missing data. These genus-level phylogenies were then midpoint rooted using the *R* package *Phangorn* (Schliep 2011). Then, using these trees, we identified all possible phylogenetically independent pairs consisting of a pathogen and nonpathogen species. This included checking that the genomes from both species were monophyletic with respect to each other, and all other species in the genus-level data set, based on the relationships of these 37 marker genes. When a nonpathogen was a sister group to multiple pathogenic species, we chose the best-sampled pathogen species with the largest number of available genomes. This process yielded the 31 pairs listed in table 1 and supplementary table S1, Supplementary Material online. We next noted a suitable outgroup for each pair, and re-estimated trees including only genomes from the pair and outgroup. These pair-level phylogenies were checked for consistency against the relevant whole-genus phylogenies and used when calculating the independent contrasts (see below).

Finally, we returned to the literature, to identify the subset of pairs with a qualitative difference in ecology between the pathogen and nonpathogen. In particular, we noted pairs where the nonpathogen was extracellular and the pathogen facultatively intracellular (pairs 2, 7, 9, 10, 12, and 23) and where the pathogen, but not the nonpathogen, replicated exclusively within their hosts in nature (pairs 3, 8, 10, 17, 23, 24, 27, and 29); these pairs are indicated in table 1.

For our *S. suis* data, we used isolates originating from six collections spanning six countries (table 2) with the same diagnostic criteria of pathogenicity status. The first collection includes isolates from the UK sampled between 2009 and 2011 (described in Weinert et al. 2015). The second includes isolates from pigs and human meningitis patients from Vietnam, sampled between 2000 and 2010 (described in Weinert et al. 2015). The third includes carriage isolates from pigs from five intensive farms in the UK, and from five intensive farms and five traditional farms in China from between 2013 and 2014 (described in Zou et al. 2018). The fourth includes disease isolates sampled from UK pigs (described in Wileman et al. 2019). The fifth includes isolates from North American and Spanish pigs, sampled between 1983 and 2016 (Hadjirin et al. 2020). The sixth includes 29 reference isolates downloaded from GenBank (NCBI). Full details of all genomes are in supplementary table S3, Supplementary Material online.

Pathogenicity status was defined in the following way. Isolates were classified as associated with “disease”, if they were recovered from systemic sites in pigs or humans with clinical signs consistent with *S. suis* infection, including meningitis, septicemia and arthritis (“systemic disease” isolates), or were recovered from a pig’s lungs in the presence of lesions of pneumonia (“respiratory disease” isolates). Isolates recovered from the tonsils or tracheo-bronchus of healthy pigs or pigs without any typical signs of *S. suis* infection were classified as “carriage”. The remaining isolates remained unclassified, due to insufficient clinical information or ambiguity in the cause of disease.

For most of the collection, serotyping was performed using antisera to known *S. suis* serotypes by the Lancefield method (Weinert et al. 2015; Wileman et al. 2019). Isolates that could not be typed with known sera were classified as nontypeable. A subset of UK isolates and the Chinese isolates were serotyped in silico using capsule genes of known serotypes (described in Zou et al. 2018). Isolates that were not serotyped were excluded from comparisons.

Sequence data from all isolates were used to generate de novo assemblies using *Spades* v.3.10.1 (Bankevich et al. 2012), after first removing low quality reads using *Sickle* v1.33 (Joshi and Fass 2011). Measures were taken to ensure all assemblies were high-quality, as described in previous studies (Weinert et al. 2015; Zou et al. 2018; Wileman et al. 2019; Hadjirin et al. 2020). Briefly, Illumina reads were mapped back to the *de novo* assembly to investigate polymorphic reads in the samples (indicative of mixed cultures) using *BWA* v.0.7.16a (Li and Durbin 2009), and genomes that exhibited poor sequencing quality (i.e., poor assembly as indicated by a large number of contigs, low N50 values or a high number of polymorphic reads) or that which were inconsistent with an *S. suis* species assignment were excluded from the analysis. Altogether this left 1,079 genomes.

To identify genetic structure in our *S. suis* isolates we identified 429 low-diversity core genes in our data set, aligned them using *DECIPHER* (Wright 2015), and stripped regions that could not be aligned unambiguously due to high divergence, indels or missing data (available on Dryad at https://doi.org/10.5061/dryad.nzs7h44qc). This conserved region of the core genome was first used to construct a consensus neighbor-joining tree using the ape package in *R* and a K80 model (Paradis et al. 2004). This tree is shown in figure 1*b* and supplementary figure S1, Supplementary Material online, and was used to generate the covariance matrices used in the phylogenetically corrected regressions (supplementary table S6 and fig. S11, Supplementary Material online). The same data were used to identify genetic clusters, using the *hierBAPS* package in *R* (Corander et al. 2008; Tonkin-Hill et al. 2018). Initial analysis identified 35 clusters. To evaluate this clustering we mapped the clusters onto the core gene phylogeny, and following the definition in Hudson et al. (1992), estimated *F_ST_* between clusters from pairwise nucleotide distances in the core gene alignment. We identified a pair of clusters with very low *F_ST_* (<0.02) that were also monophyletic in the tree, and these clusters were combined to form cluster 3 (supplementary table S1, Supplementary Material online). Full details of all clusters are found in supplementary table S4, Supplementary Material online.

The illustrative genealogy shown in figure 1*c* involved mapping to the reference genome BM407 (see supplementary table S3, Supplementary Material online) using *Bowtie2* (Langmead and Salzberg 2012), recombination-stripping in *Gubbins* (Croucher et al. 2015), and tree construction with *MrBayes* (Huelsenbeck and Ronquist 2001) with default parameters and the HKY + Γ substitution model.

### Genome Annotation

For between-species data, we used the *RefSeq* annotations. We also carried out re-annotations using *Prokka* (v2.8.2) (Seemann 2014). Although *Prokka* and *RefSeq* annotations were generally congruent, *Prokka* does not explicitly annotate pseudogenes and thus high levels of pseudogenization in a handful of species (e.g., *Rickettsia prowazekii*), led to erratic results, so we preferred *RefSeq* annotations. We also excluded plasmids because these can be lost during culture and sequencing. However, the main results concerning genome size are robust to their inclusion (supplementary table S5, Supplementary Material online).

The draft *S. suis* genomes were also annotated using *Prokka* (v2.8.2) (Seemann 2014). Orthologous genes were initially identified using *Roary* (Page et al. 2015), with the recommended parameter values. We then manually curated these orthology groups, in order to identify orthologous genes that had been wrongly placed in distinct orthology groups either due to high levels of divergence or incomplete assemblies. We also checked all instances of gene absence in each orthology group, since these might have resulted from incomplete genome assemblies. This was undertaken using all-against-all gene group nucleotide *BLAST* search (*BLASTN*), and *BLASTN* search of all orthology groups against all of the genomes in which that group was described as absent (Camacho et al. 2013). The final set of orthology groups were used to define the core genome (supplementary figs. S7 and S8, Supplementary Material online).

In figure 3 and related analysis, we defined the “functional” proportion of the genome as any region annotated as a protein- or RNA-coding locus. For both data sets, all protein-coding genes were assigned a COG category, and categories C, E, F, G, H, I, P, and Q defined as “metabolic genes”. For the *S. suis* data set, we also identified genes that were annotated as transposases or integrases in the *Prokka* annotations. For the 29 *S. suis* isolates with complete assemblies we identified mobile genetic elements using IslandViewer 4 (Bertelli et al. 2017).

### Statistical Analyses

For the between-species data, each comparison pair differed in the number of genomes sampled, and the amount of evolutionary change between the species. For this reason, we standardized the weightings using a method of independent contrasts. In brief, each comparison point was equivalent to the difference between the ancestral trait values for the sampled genomes from the pathogenic and nonpathogenic species that would be inferred from a Brownian motion model of trait evolution (and using the tip value in the case of a single genome). The contrast for each pair was then standardized by its associated standard deviation. The method used the pic and ace functions in the *ape* package in *R* (Felsenstein 1985; Paradis et al. 2004), and for each pair, we used the genealogies constructed from the 37 “universal genes”, described above, so that amounts of molecular evolution were comparable across the entire data set. We also added a fixed constant of 1/length(alignment) to deal with zero-length branches in some of the genealogies (reducing this constant by a factor of 10 had no appreciable effect on our results). For each variable, we then applied a standard transformation. We chose a logarithmic transformation for data that are positively valued but unbounded above (such as genome size and gene number), so that doublings in value were always represented as changes of the same size. For data that are proportions, such as GC content, and are therefore bounded at 0 and 1, we used a logit transformation, log(*x*/(1–*x*)). To test the suitability of these transformations, and to understand the general patterns of evolution in each variable, we tested the validity of the Brownian motion model following the recommendations of (Freckleton 2000). As shown in supplementary figures S4 and S6, Supplementary Material online, for most traits, the model provided a good fit. The sole exception was the proportion of genomes with known function (supplementary fig. S6*d*–*f*, Supplementary Material online), which is consistent with the rapid loss of nonfunctional elements, as discussed in the main text. Even after standardizing the variances, the set of contrasts was usually highly nonnormal (e.g., fig. 2*a*), and so we tested the null hypothesis of a vanishing mean (i.e., no consistent trait difference between pathogenic and nonpathogenic species) by randomly permuting the labels “pathogen” and “nonpathogen” within each pair (i.e., randomly choosing the sign of each of the 31 contrasts). The test statistic was the mean contrast value (using the true signs), and 10^6^ random permutations were used to construct its null distribution. We also repeated results after removing outliers, identified by eye (supplementary table S5, Supplementary Material online). The same permutation approach was used for the within-cluster data set, although here, the disease and carriage isolates were interspersed in the genealogy, and so we used the raw means of the trait values for each class of isolate.

For the between-cluster analyses, tests also had to account for the differences in cluster size. For this reason, most results used weighted linear regression (using the square root of the number of isolates in each cluster as weights). Because these analyses ignored possible covariances between clusters, due to their shared ancestry, we also used phylogenetically corrected regressions, retaining only the larger clusters (containing at least 20 isolates). These analyses, shown in supplementary table S6, Supplementary Material online data set “D,” used the *gls* function in the *nlme* package in *R* (Pinheiro et al. 2015), and Pagel’s “lambda correlation structure” (*corPagel* in the *ape* package, Pagel 1999; Paradis et al. 2004).

## Supplementary Material

Supplementary data are available at *Molecular Biology and Evolution* online.

## Supplementary Material

msaa323_Supplementary_Data

## References

[msaa323-B1] Baig A, Weinert LA, Peters SE, Howell KJ, Chaudhuri RR, Wang J, Holden MTG, Parkhill J, Langford PR, Rycroft AN, et al 2015. Whole genome investigation of a divergent clade of the pathogen *Streptococcus suis*. Front Microbiol. 6:1191. 26583006 10.3389/fmicb.2015.01191 PMC4631834

[msaa323-B2] Bankevich A, Nurk S, Antipov D, Gurevich AA, Dvorkin M, Kulikov AS, Lesin VM, Nikolenko SI, Pham S, Prjibelski AD, et al 2012. SPAdes: a new genome assembly algorithm and its applications to single-cell sequencing. J Comput Biol. 19(5):455–477. 22506599 10.1089/cmb.2012.0021 PMC3342519

[msaa323-B3] Bertelli C, Laird MR, Williams KP, Research Computing Group SFU, Lau BY, Hoad GL, Winsor GL, Brinkman FSL, 2017. IslandViewer 4: expanded prediction of genomic islands for larger-scale datasets. Nucleic Acids Res. 45(W1):W30–W35. 28472413 10.1093/nar/gkx343 PMC5570257

[msaa323-B5] Bobay L-M, Ochman H. 2018. Factors driving effective population size and pan-genome evolution in bacteria. BMC Evol Biol. 18(1):153. 30314447 10.1186/s12862-018-1272-4 PMC6186134

[msaa323-B6] Camacho C, Madden T, Ma N, Tao T, Agarwala R, Morgulis A. 2013. BLAST command line applications user manual. Boca Raton (FL).

[msaa323-B7] Corander J, Marttinen P, Sirén J, Tang J. 2008. Enhanced Bayesian modelling in BAPS software for learning genetic structures of populations. BMC Bioinformatics 9(1):539. 19087322 10.1186/1471-2105-9-539 PMC2629778

[msaa323-B8] Croucher NJ, Page AJ, Connor TR, Delaney AJ, Keane JA, Bentley SD, Parkhill J, Harris SR. 2015. Rapid phylogenetic analysis of large samples of recombinant bacterial whole genome sequences using Gubbins. Nucleic Acids Res. 43(3):e15. 25414349 10.1093/nar/gku1196 PMC4330336

[msaa323-B9] Darling AE, Jospin G, Lowe E, Matsen FA, Bik HM, Eisen JA. 2014. PhyloSift: phylogenetic analysis of genomes and metagenomes. PeerJ 2:e243. 24482762 10.7717/peerj.243 PMC3897386

[msaa323-B10] Dietel A-K, Merker H, Kaltenpoth M, Kost C. 2019. Selective advantages favour high genomic AT-contents in intracellular elements. PLoS Genet. 15(4):e1007778. 31034469 10.1371/journal.pgen.1007778 PMC6519830

[msaa323-B11] Felsenstein J. 1985. Phylogenies and the comparative method. Am Nat. 125(1):1–15.

[msaa323-B12] Freckleton RP. 2000. Phylogenetic tests of ecological and evolutionary hypotheses: checking for phylogenetic independence. Funct Ecol. 14(1):129–134.

[msaa323-B13] Georgiades K, Raoult D. 2011. Genomes of the most dangerous epidemic bacteria have a virulence repertoire characterized by fewer genes but more toxin-antitoxin modules. PLoS ONE 6(3):e17962. 21437250 10.1371/journal.pone.0017962 PMC3060909

[msaa323-B14] Hadjirin NF, Miller EL, Murray GGR, Yen PLK, Phuc HD, Wileman TM, Hernandez-Garcia J, Williamson SM, Parkhill J, Maskell DJ, et al. 2020. Linking phenotype, genotype and ecology: Antimicrobial resistance in the zoonotic pathogen *Streptococcus suis*. bioRxiv; doi:10.1101/2020.05.05.078493. 10.1186/s12915-021-01094-1 PMC8422772 34493269

[msaa323-B15] Hershberg R, Petrov DA. 2010. Evidence that mutation is universally biased towards AT in bacteria. PLoS Genet. 6(9):e1001115. 20838599 10.1371/journal.pgen.1001115 PMC2936535

[msaa323-B16] Hildebrand F, Meyer A, Eyre-Walker A. 2010. Evidence of selection upon genomic GC-content in bacteria. PLoS Genet. 6(9):e1001107. 20838593 10.1371/journal.pgen.1001107 PMC2936529

[msaa323-B17] Huang SS, Hinrichsen VL, Datta R, Spurchise L, Miroshnik I, Nelson K, Platt R. 2011. Methicillin-resistant *Staphylococcus aureus* infection and hospitalization in high-risk patients in the year following detection. PLoS ONE 6(9):e24340. 21949707 10.1371/journal.pone.0024340 PMC3174953

[msaa323-B18] Hudson RR, Slatkin M, Maddison WP. 1992. Estimation of levels of gene flow from DNA sequence data. Genetics 132(2):583–589. 1427045 10.1093/genetics/132.2.583 PMC1205159

[msaa323-B19] Huelsenbeck JP, Ronquist F. 2001. MRBAYES: Bayesian inference of phylogenetic trees. Bioinformatics 17(8):754–755. 11524383 10.1093/bioinformatics/17.8.754

[msaa323-B20] Joshi NA, Fass JN. 2011. Sickle: a sliding-window, adaptive, quality-based trimming tool for FastQ files. Available from: https://github.com/najoshi/sickle.

[msaa323-B21] Kono M, Zafar MA, Zuniga M, Roche AM, Hamaguchi S, Weiser JN. 2016. Single cell bottlenecks in the pathogenesis of *Streptococcus pneumoniae*. PLoS Pathog. 12(10):e1005887. 27732665 10.1371/journal.ppat.1005887 PMC5061371

[msaa323-B22] Kumar S, Stecher G, Tamura K. 2016. MEGA7: molecular evolutionary genetics analysis version 7.0 for bigger datasets. Mol Biol Evol. 33(7):1870–1874. 27004904 10.1093/molbev/msw054 PMC8210823

[msaa323-B23] Kuo C-H, Moran NA, Ochman H. 2009. The consequences of genetic drift for bacterial genome complexity. Genome Res. 19(8):1450–1454. 19502381 10.1101/gr.091785.109 PMC2720180

[msaa323-B24] Langmead B, Salzberg SL. 2012. Fast gapped-read alignment with Bowtie 2. Nat Methods. 9(4):357–359. 22388286 10.1038/nmeth.1923 PMC3322381

[msaa323-B25] Langridge GC, Fookes M, Connor TR, Feltwell T, Feasey N, Parsons BN, Seth-Smith HMB, Barquist L, Stedman A, Humphrey T, et al 2015. Patterns of genome evolution that have accompanied host adaptation in *Salmonella*. Proc Natl Acad Sci USA. 112(3):863–868. 25535353 10.1073/pnas.1416707112 PMC4311825

[msaa323-B26] Lassalle F, Perian S, Bataillon T, Nesme X, Duret L, Daubin V. 2015. GC-content evolution in bacterial genomes: the biased gene conversion hypothesis expands. PLoS Genet. 11(2):e1004941. 25659072 10.1371/journal.pgen.1004941 PMC4450053

[msaa323-B27] Li H, Durbin R. 2009. Fast and accurate short read alignment with Burrows–Wheeler transform. Bioinformatics 25(14):1754–1760. 19451168 10.1093/bioinformatics/btp324 PMC2705234

[msaa323-B28] McCutcheon JP, Moran NA. 2007. Parallel genomic evolution and metabolic interdependence in an ancient symbiosis. Proc Natl Acad Sci USA. 104(49):19392–19397. 18048332 10.1073/pnas.0708855104 PMC2148300

[msaa323-B29] McCutcheon JP, Moran NA. 2012. Extreme genome reduction in symbiotic bacteria. Nat Rev Microbiol. 10(1):13–26. 10.1038/nrmicro2670 22064560

[msaa323-B30] Mira A, Ochman H, Moran NA. 2001. Deletional bias and the evolution of bacterial genomes. Trends Genet. 17(10):589–596. 11585665 10.1016/s0168-9525(01)02447-7

[msaa323-B31] Moran NA. 2002. Microbial minimalism. Cell 108(5):583–586. 11893328 10.1016/s0092-8674(02)00665-7

[msaa323-B32] Nakabachi A, Yamashita A, Toh H, Ishikawa H, Dunbar HE, Moran NA, Hattori M. 2006. The 160-kilobase genome of the bacterial endosymbiont *Carsonella*. Science 314(5797):267–267. 17038615 10.1126/science.1134196

[msaa323-B34] Newton ILG, Bordenstein SR. 2011. Correlations between bacterial ecology and mobile DNA. Curr Microbiol. 62(1):198–298. 20577742 10.1007/s00284-010-9693-3 PMC3006647

[msaa323-B35] Ochman H, Moran NA. 2001. Genes lost and genes found: evolution of bacterial pathogenesis and symbiosis. Science 292(5519):1096–1099. 11352062 10.1126/science.1058543

[msaa323-B36] O’Leary NA, Wright MW, Brister JR, Ciufo S, Haddad D, McVeigh R, Rajput B, Robbertse B, Smith-White B, Ako-Adjei D, et al 2016. Reference sequence (RefSeq) database at NCBI: current status, taxonomic expansion, and functional annotation. Nucleic Acids Res. 44(D1):D733–D745. 26553804 10.1093/nar/gkv1189 PMC4702849

[msaa323-B37] Page AJ, Cummins CA, Hunt M, Wong VK, Reuter S, Holden MTG, Fookes M, Falush D, Keane JA, Parkhill J. 2015. Roary: rapid large-scale prokaryote pan genome analysis. Bioinformatics 31(22):3691–3693. 26198102 10.1093/bioinformatics/btv421 PMC4817141

[msaa323-B38] Pagel M. 1999. Inferring the historical patterns of biological evolution. Nature 401(6756):877–884. 10553904 10.1038/44766

[msaa323-B39] Paradis E, Claude J, Strimmer K. 2004. APE: analyses of phylogenetics and evolution in R language. Bioinformatics 20(2):289–290. 14734327 10.1093/bioinformatics/btg412

[msaa323-B40] Pinheiro J, Bates D, DebRoy S, Sarkar D, R-core 2015. nlme: linear and nonlinear mixed effects models. Available from: http://cran.r-project.org/web/packages/nlme/index.html.

[msaa323-B41] Pupo GM, Lan R, Reeves PR. 2000. Multiple independent origins of *Shigella* clones of *Escherichia coli* and convergent evolution of many of their characteristics. Proc Natl Acad Sci USA. 97(19):10567–10572. 10954745 10.1073/pnas.180094797 PMC27065

[msaa323-B42] Rao Q, Rollat-Farnier P-A, Zhu D-T, Santos-Garcia D, Silva FJ, Moya A, Latorre A, Klein CC, Vavre F, Sagot M-F, et al 2015. Genome reduction and potential metabolic complementation of the dual endosymbionts in the whitefly *Bemisia tabaci*. BMC Genomics 16(1):226. 25887812 10.1186/s12864-015-1379-6 PMC4438442

[msaa323-B43] Rautelin H, Hänninen ML, Sivonen A, Turunen U, Valtonen V. 1995. Chronic diarrhea due to a single strain of *Aeromonas caviae*. Eur J Clin Microbiol Infect Dis. 14(1):51–53. 7537217 10.1007/BF02112620

[msaa323-B44] Rocha EPC, Danchin A. 2002. Base composition bias might result from competition for metabolic resources. Trends Genet. 18(6):291–294. 12044357 10.1016/S0168-9525(02)02690-2

[msaa323-B45] Schliep KP. 2011. phangorn: phylogenetic analysis in R. Bioinformatics 27(4):592–593. 21169378 10.1093/bioinformatics/btq706 PMC3035803

[msaa323-B46] Seemann T. 2014. Prokka: rapid prokaryotic genome annotation. Bioinformatics 30(14):2068–2069. 24642063 10.1093/bioinformatics/btu153

[msaa323-B47] Sheppard SK, Guttman DS, Ross Fitzgerald J. 2018. Population genomics of bacterial host adaptation. Nat Rev Genet. 19(9):549–565. 29973680 10.1038/s41576-018-0032-z

[msaa323-B48] Stinear TP, Seemann T, Harrison PF, Jenkin GA, Davies JK, Johnson PDR, Abdellah Z, Arrowsmith C, Chillingworth T, Churcher C, et al 2008. Insights from the complete genome sequence of *Mycobacterium marinum* on the evolution of *Mycobacterium tuberculosis*. Genome Res. 18(5):729–741. 18403782 10.1101/gr.075069.107 PMC2336800

[msaa323-B49] Toft C, Andersson SGE. 2010. Evolutionary microbial genomics: insights into bacterial host adaptation. Nat Rev Genet. 11(7):465–475. 20517341 10.1038/nrg2798

[msaa323-B50] Tonkin-Hill G, Lees JA, Bentley SD, Frost SDW, Corander J. 2018. RhierBAPS: an R implementation of the population clustering algorithm hierBAPS. Wellcome Open Res. 3:93. 30345380 10.12688/wellcomeopenres.14694.1 PMC6178908

[msaa323-B51] Vötsch D, Willenborg M, Weldearegay YB, Valentin-Weigand P. 2018. *Streptococcus suis*—the “two faces” of a pathobiont in the porcine respiratory tract. Front Microbiol. 9:480. 29599763 10.3389/fmicb.2018.00480 PMC5862822

[msaa323-B52] Vouga M, Greub G. 2016. Emerging bacterial pathogens: the past and beyond. Clin Microbiol Infect. 22(1):12–21. 26493844 10.1016/j.cmi.2015.10.010 PMC7128729

[msaa323-B53] Weinert LA, Chaudhuri RR, Wang J, Peters SE, Corander J, Jombart T, Baig A, Howell KJ, Vehkala M, Välimäki N, et al 2015. Genomic signatures of human and animal disease in the zoonotic pathogen *Streptococcus suis*. Nat Commun. 6:6740. 25824154 10.1038/ncomms7740 PMC4389249

[msaa323-B54] Weinert LA, Welch JJ. 2017. Why might bacterial pathogens have small genomes? Trends Ecol Evol. 32(12):936–947. 29054300 10.1016/j.tree.2017.09.006

[msaa323-B55] Whitman WB. ed. 2015. Bergey’s manual of systematics of archaea and bacteria. Wiley.

[msaa323-B56] Wileman TM, Weinert LA, Howell KJ, Wang J, Peters SE, Williamson SM, Wells JM, Langford PR, Rycroft AN, Wren BW, et al 2019. Pathotyping the zoonotic pathogen *Streptococcus suis*: novel genetic markers to differentiate invasive disease-associated isolates from non-disease-associated isolates from England and Wales. J Clin Microbiol. 57:e01712–18. 30944194 10.1128/JCM.01712-18 PMC6595460

[msaa323-B57] Wright ES. 2015. DECIPHER: harnessing local sequence context to improve protein multiple sequence alignment. BMC Bioinformatics 16(1):322. 26445311 10.1186/s12859-015-0749-z PMC4595117

[msaa323-B58] Zientz E, Dandekar T, et al 2004. Metabolic interdependence of obligate intracellular bacteria and their insect hosts. Microbiol Mol Biol Rev. 68(4):745–70. 15590782 10.1128/MMBR.68.4.745-770.2004 PMC539007

[msaa323-B59] Zhu Q, Mai U, Pfeiffer W, Janssen S, Asnicar F, Sanders JG, Belda-Ferre P, Al-Ghalith GA, Kopylova E, McDonald D, et al 2019. Phylogenomics of 10,575 genomes reveals evolutionary proximity between domains Bacteria and Archaea. Nat Commun. 10(1):5477. 31792218 10.1038/s41467-019-13443-4 PMC6889312

[msaa323-B60] Zou G, Zhou J, Xiao R, Zhang L, Cheng Y, Jin H, Li L, Zhang L, Wu B, Qian P, et al 2018. Effects of environmental and management-associated factors on prevalence and diversity of *Streptococcus suis* in clinically healthy pig herds in China and the United Kingdom. Appl Environ Microbiol. 84(8):e02590–17. 29427423 10.1128/AEM.02590-17 PMC5881051

