# Supplementary material for: Genome Reduction Is Associated with Bacterial Pathogenicity across Different Scales of Temporal and Ecological Divergence
Source: Mol Biol Evol. 2020 Dec 12;38(4):1570–9. doi: 10.1093/molbev/msaa323 (PMC8042751; doi:10.1093/molbev/msaa323)
Supplement: msaa323_Supplementary_Data [file msaa323_Supplementary_Data.zip › Murray & Charlesworth et al. - Supplementary Figures.pdf]

Supplementary figures for

Genome reduction is associated with bacterial pathogenicity across different scales of temporal and ecological divergence

Gemma G. R. Murray, Jane Charlesworth, Eric L. Miller, Michael J. Casey, Catrin T. Lloyd, Marcelo Gottschalk, A. W. (Dan) Tucker, John J. Welch, and Lucy A. Weinert

Corresponding authors: Gemma G. R. Murray & Jane Charlesworth
Emails: ggrmurray@gmail.com; janepipistrelle@googlemail.com

This PDF file includes:

Figures S1 to S12
SI References

Other supplementary materials for this manuscript include the following:

Tables S1 to S7

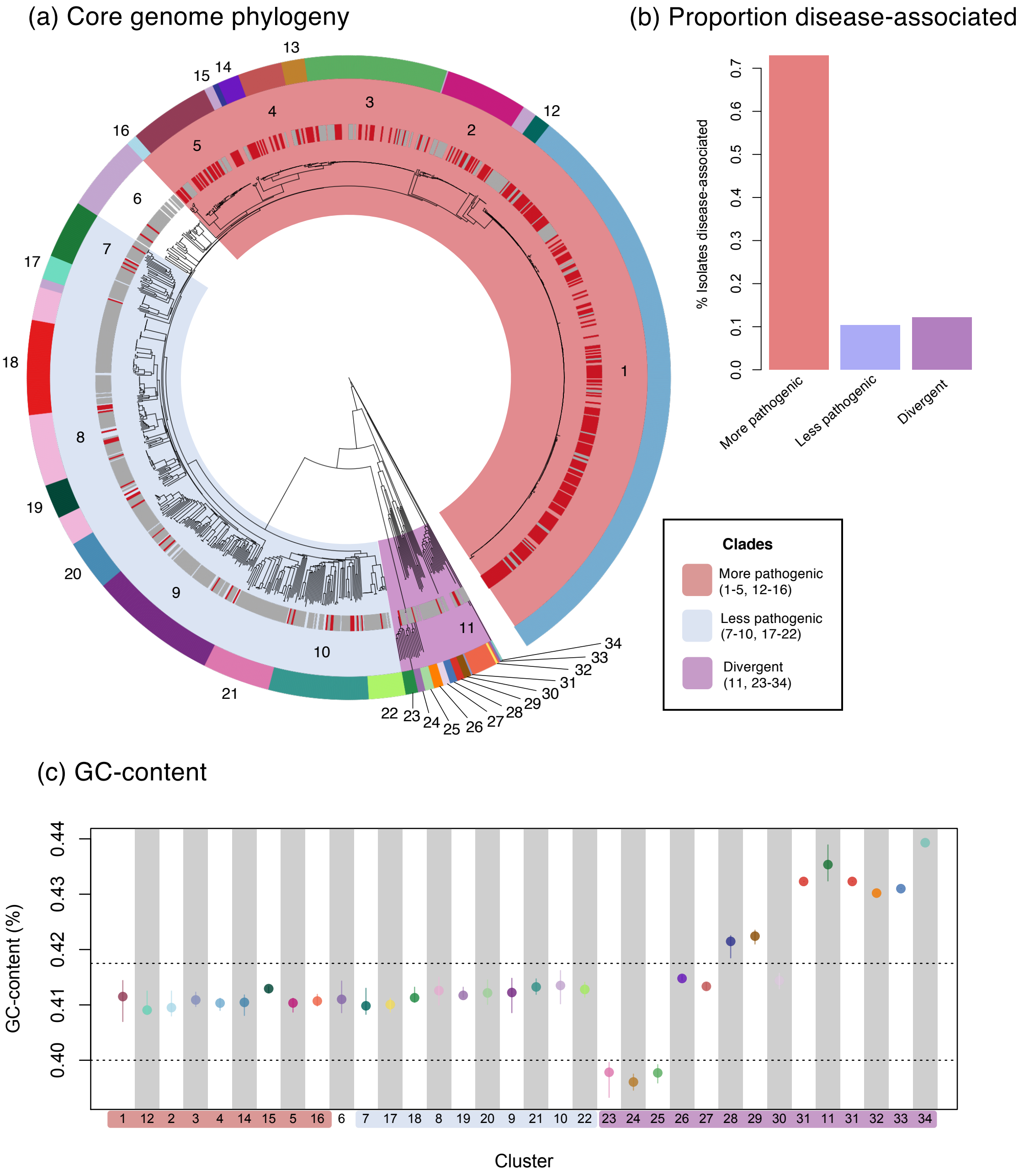

Fig. S1. The genetic structure of *S. suis*. (a) A core genome phylogeny of our 1079 isolates of *S. suis*. Individual disease (red) and carriage (grey) isolates are indicated in the inner strip. The outermost strip describes the 34 genetic clusters. The 11 "mixed clusters" that include multiple disease and carriage isolates are numbered within the strip (clusters 1-11), and the other 23 clusters are numbered outside the strip (clusters 12-34). The 'more pathogenic' clade is highlighted in red, the 'less pathogenic' clade in blue, and the 'divergent' clade in purple. (b) The proportion of isolates in each clade that are associated with disease (not including isolates for which disease-association is unknown). (c) The whole-genome GC-content for each cluster, with the three clades highlighted. Dashed lines indicate the range of GC values that was used to exclude 10 outlying clusters (3 with low GC, and 7 with high GC), in Figures S7b and S8.

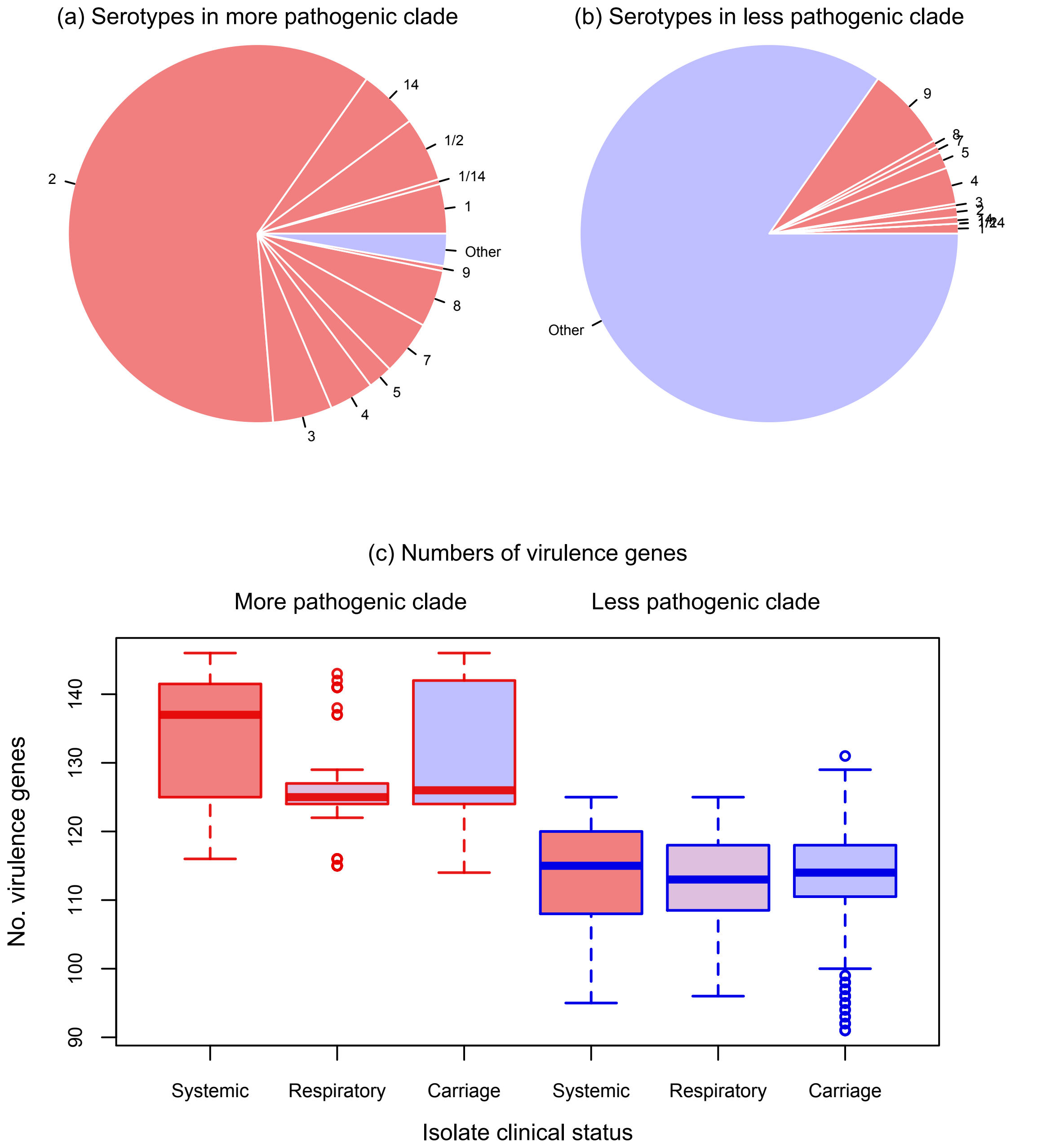

Fig. S2. The reliability of disease-associated phenotyping in the *S. suis* data. (a) Pie charts showing the proportion of disease-associated serotypes (serotypes 2, 1/2, 1, 14, 3, 4, 5, 7, 8, 9) (Segura et al. 2017, Prüfer et al. 2019) present in our more and less pathogenic clades. (b) Box plots showing the numbers of virulence genes present in isolates from our more and less pathogenic clades, and in isolates with different clinical statuses within these clades. This is based on a set of 157 virulence genes that were identified in several previous studies of *S. suis* virulence (Segura et al. 2017, Fittipaldi et al. 2012, Holden et al. 2009, Wilson et al. 2007, Zheng et al. 2012).

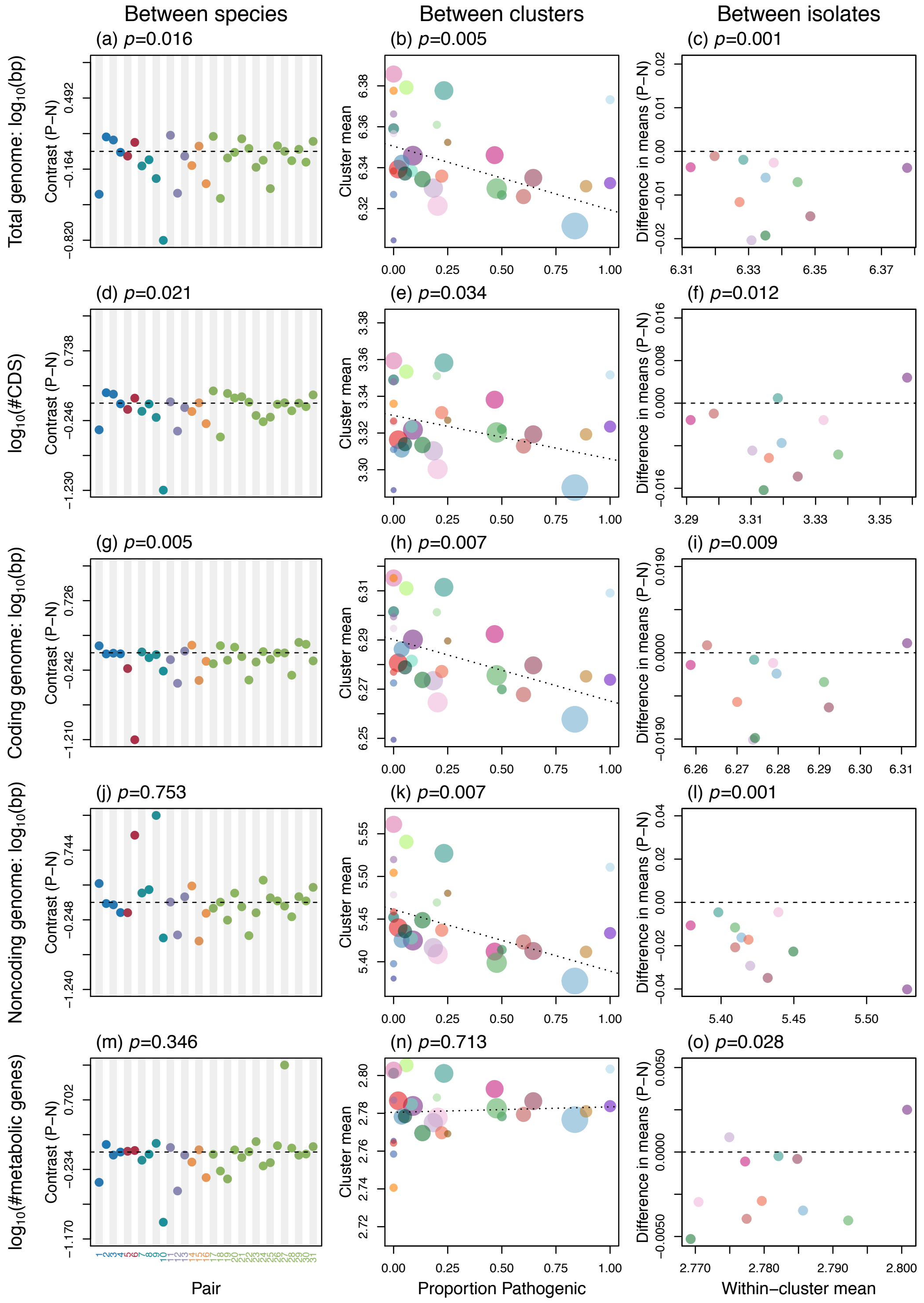

Fig. S3. The association of pathogenicity with different measures of genome size (a-i), and the endosymbiont syndrome (j-o). (a-c) Total genome size after including plasmids (note that this makes no difference for the *Ssuis* data set); (d-f) Total number of intact protein-coding genes; (g-i) Total length of protein and RNA-coding elements; (j-l): Length of genome non coding for proteins or RNAs; (m-o) The number of intact protein-coding genes with metabolic function. All other details match Figure 2.

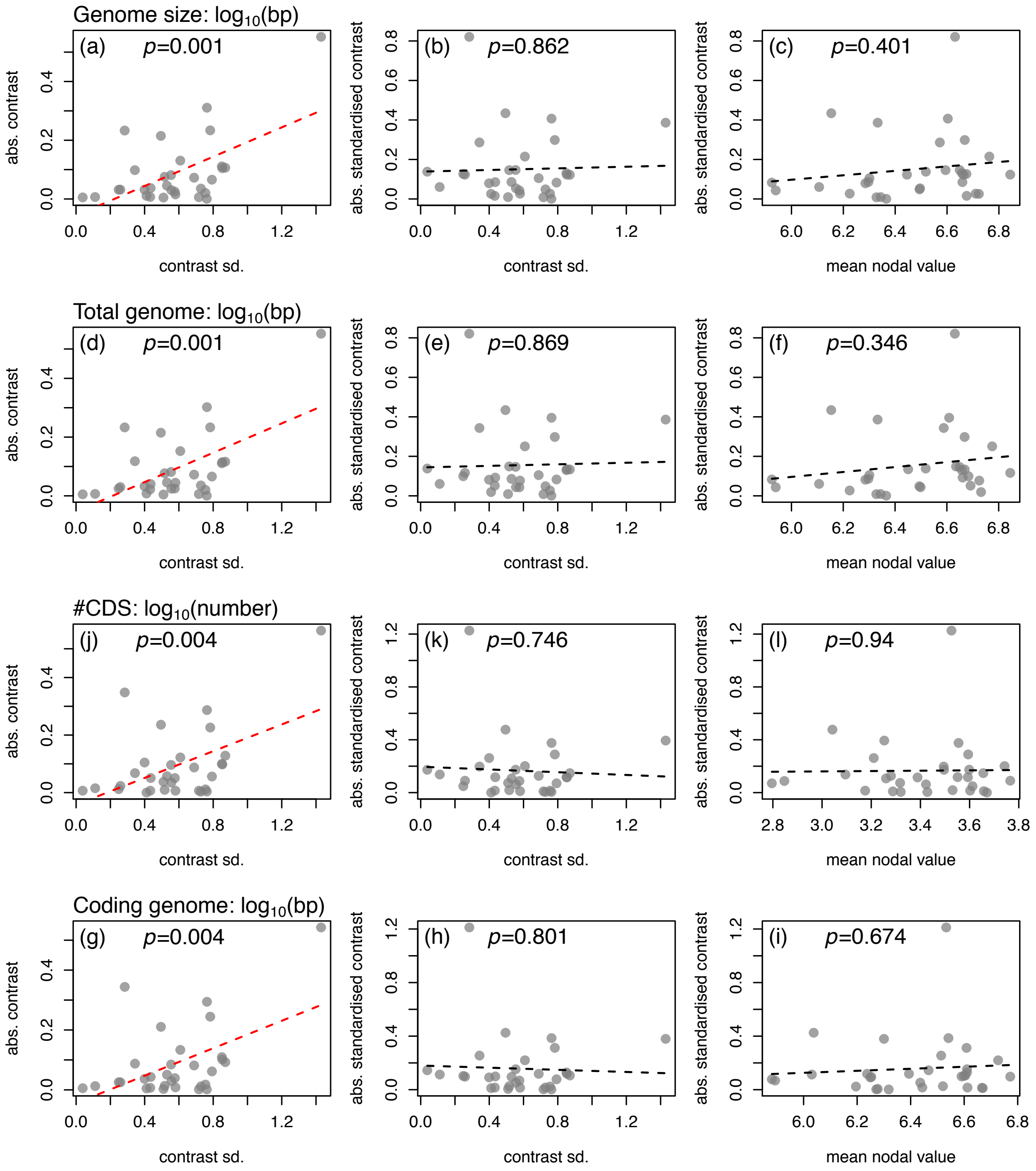

Fig. S4. Adequacy of the Brownian Motion model used to generate between-species independent contrasts for genome size. The left-hand column plots the absolute values of the raw (unstandardised contrasts) against the contrast standard deviations, as inferred from the trait values and bacterial genealogy. A significant positive regression slope is consistent with the Brownian Motion model. The middle and right-hand columns plot the absolute value of the standardised contrast against its standard deviation, or the mean trait value for the two relevant nodes of the genealogy. The lack of a relationship, in each case, is consistent with the Brownian Motion model, and implies that all points should have equal weight in the tests. Each column corresponds to a different measure of genome size, matching those used in Figure 2 (a-c), or Figure S3 (d-i).

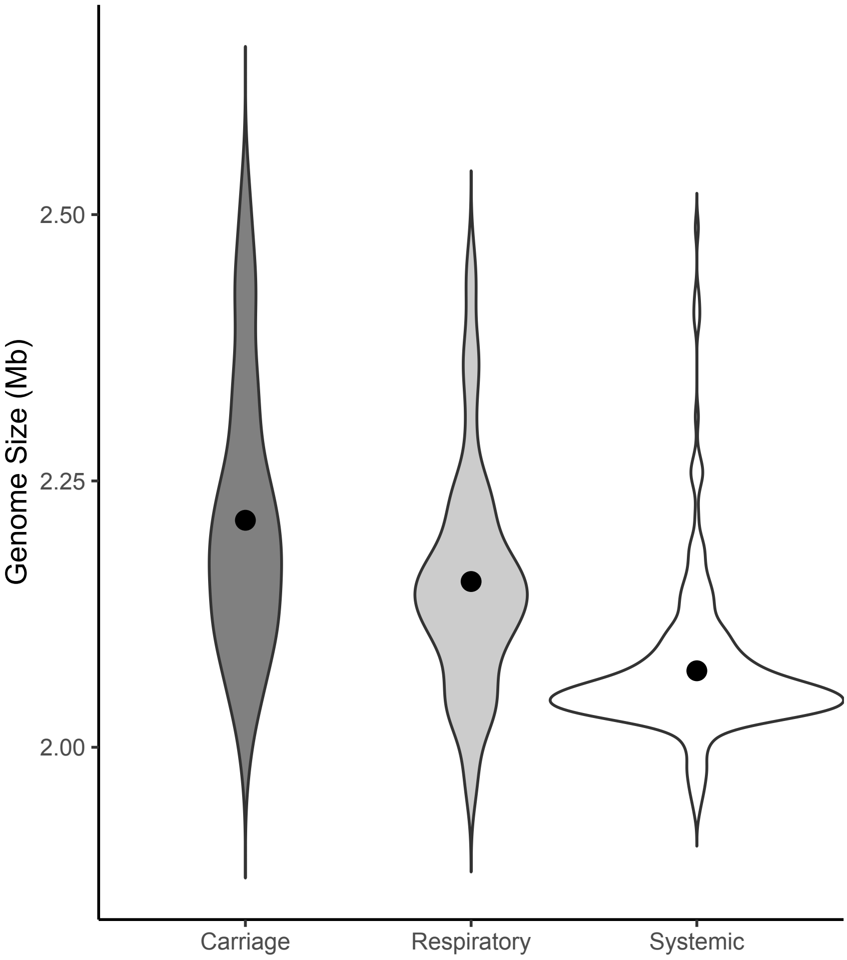

Fig. S5. Genome size and disease severity in *S. suis.* Violin plots of the distribution of genome sizes for the three classes of *S. suis* isolates used in this study. Across the data set as a whole, there is a tendency for the most serious systemic disease isolates to have the smallest genomes, while isolates associated with less serious respiratory disease have genomes of intermediate size.

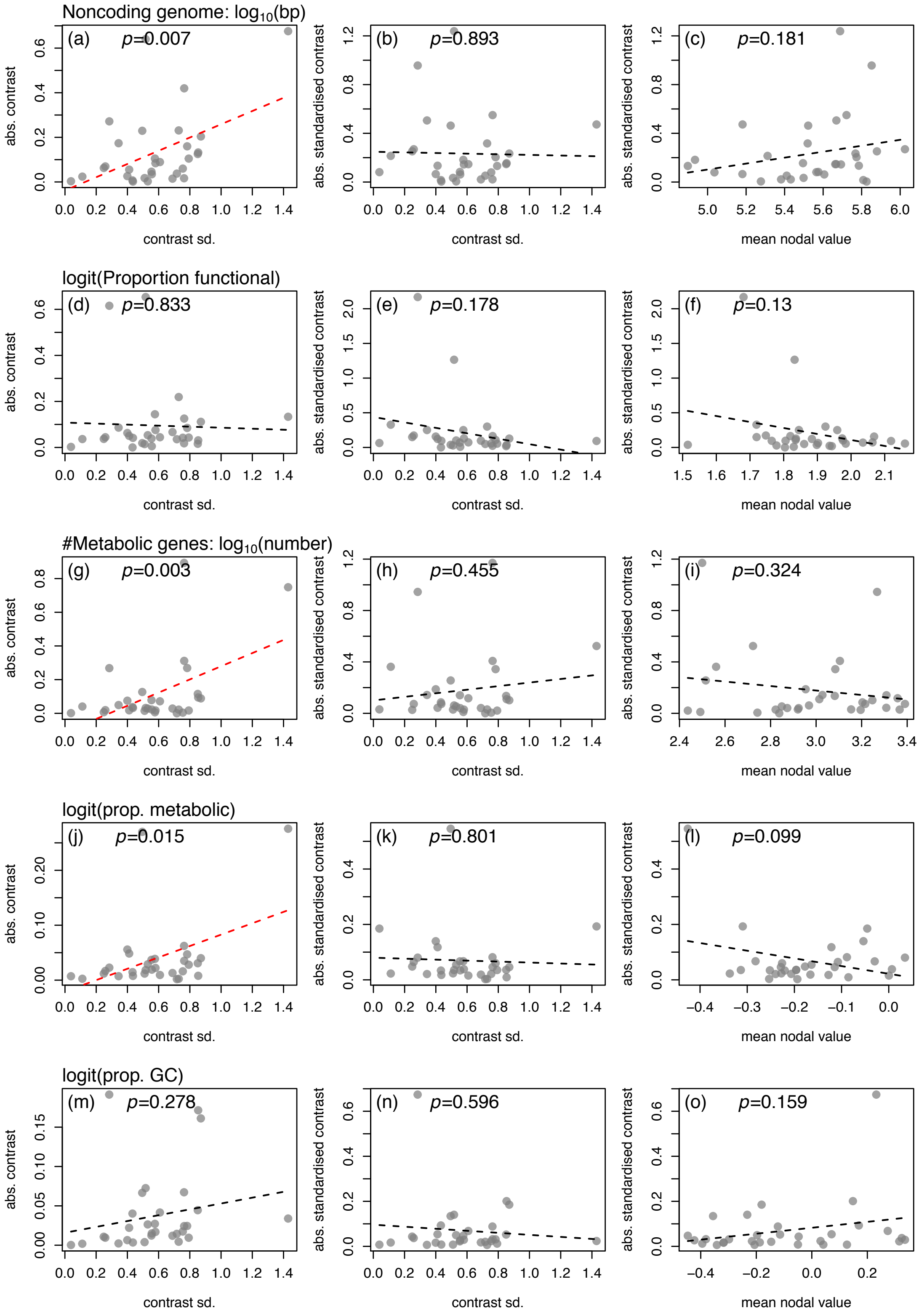

Fig. S6. Adequacy of the Brownian Motion model used to generate between-species independent contrasts for measures of the endosymbiont syndrome. All details match Figure S4, except for the trait values used, which match those in Figure 3 (d-f, j-o) or Figure S3 (a-c, g-i). Of note is the failure of the Brownian motion model to fit the proportion of the genome that is functional (d-f).

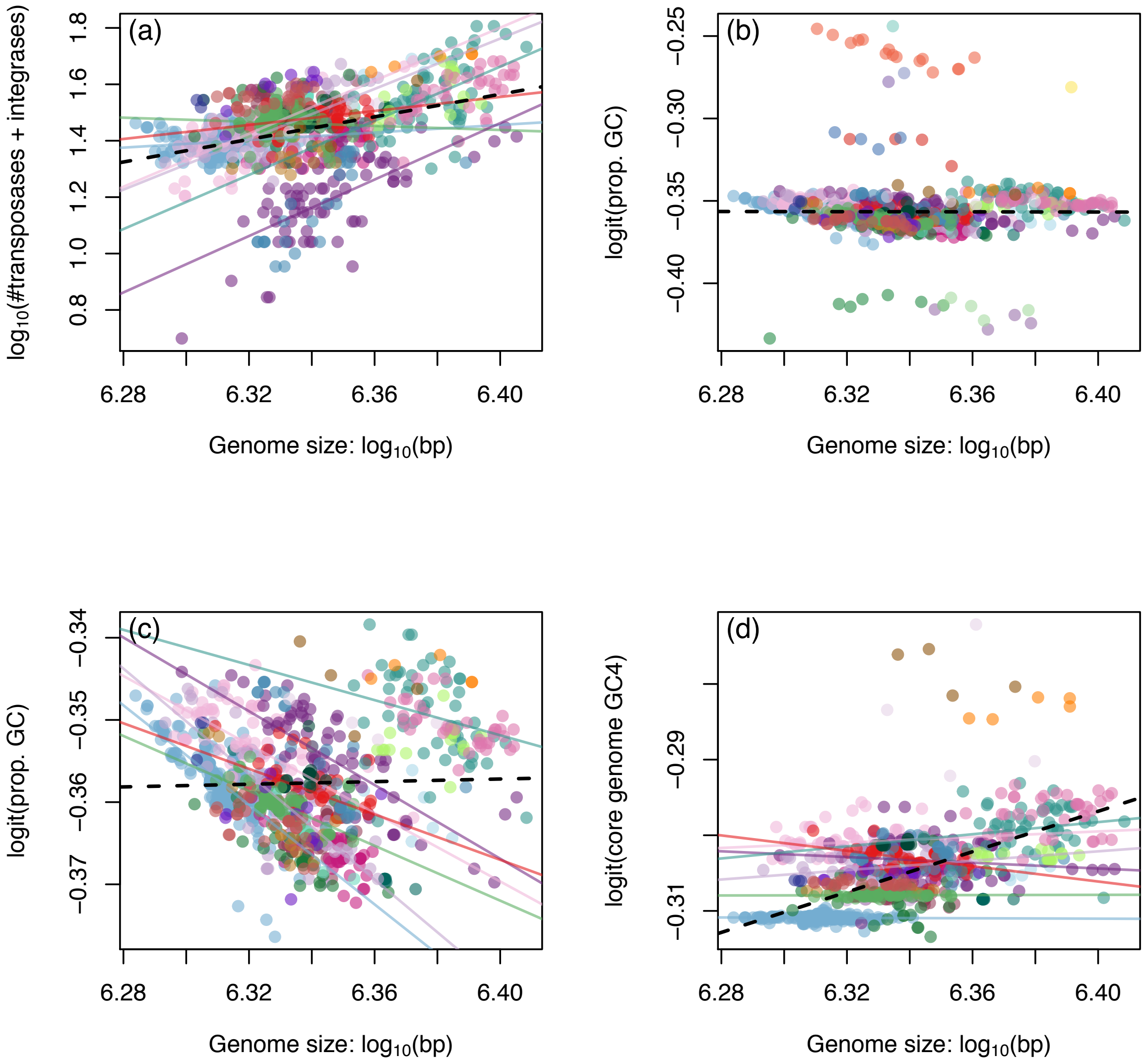

Fig. S7. Variation in genomic GC content within the *S. suis* data. Each point represents a single *S. suis* isolate, coloured according to the genetic clusters from Figure 1b, and all lines represent best-fit regression slopes using ordinary least-squares. (a) Evidence of the rapid dynamics of mobile elements, with transposes and integrases more common in isolates with larger genomes. While there is a positive correlation between the number of these genes and genome size across the data as a whole (dashed line), there are even clearer trends within each of the clusters (coloured lines show results for the 7 largest clusters). (b) Interpreting the relationship between GC-content and genome size is complicated by divergent clusters with anomalous GC values (see Figure S1). (c) After removing these clusters, there is not clear association in the data as a whole (dashed line), but clear negative trends within each of the clusters (coloured solid lines). (d) These patterns change when we consider only sites in the core genome (i.e., sites present in all isolates). In particular, for the most rapidly evolving fourfold degenerate sites in protein coding genes, there is little variation in GC4 within clusters (coloured solid lines), but a positive relationship across the data as whole (black dashed line).

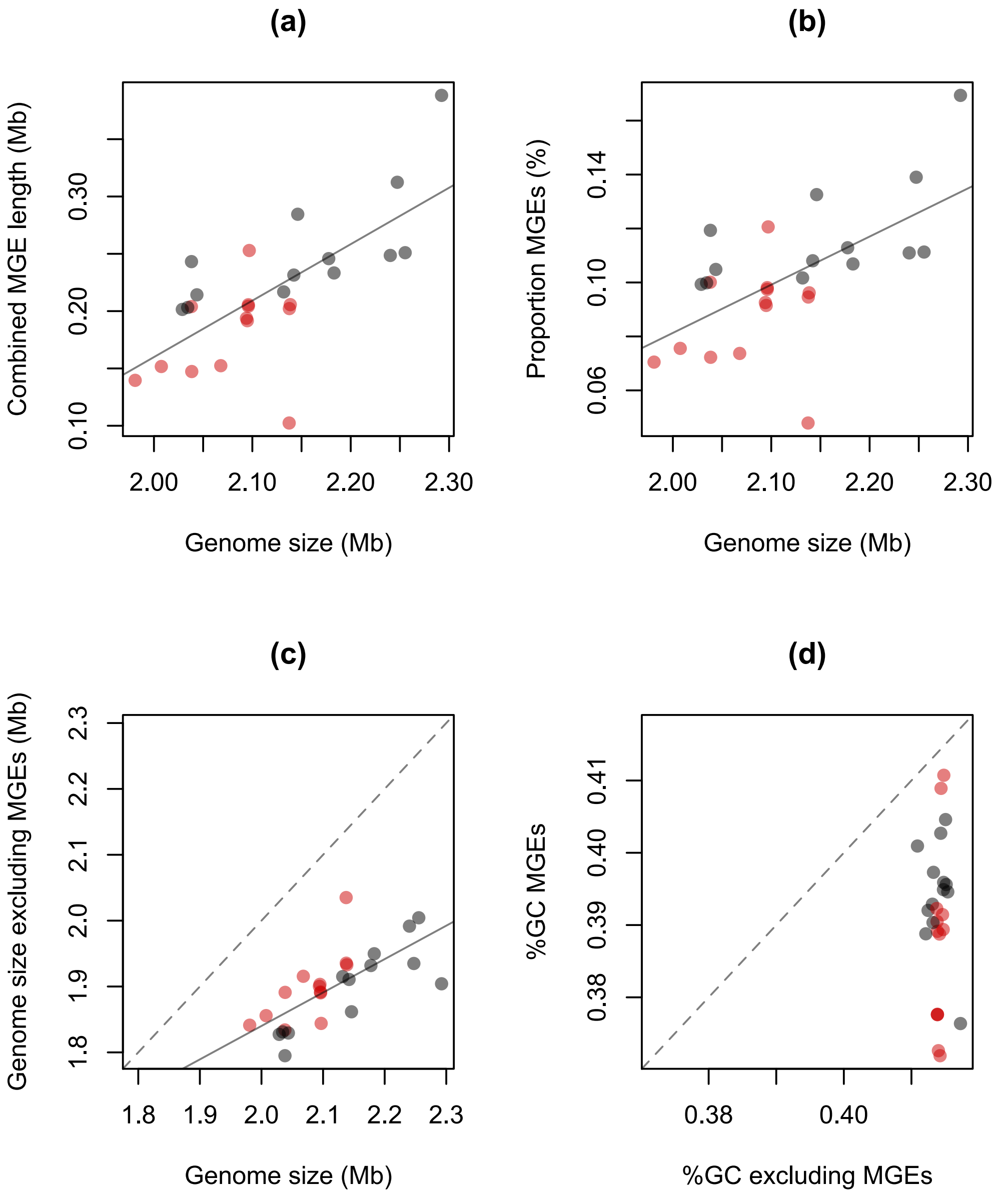

Fig. S8. The contribution of mobile genetic elements (MGEs) to the variation in genome size in *S. suis*. Estimates of the size and GC-content of mobile elements are shown for each of the 29 *S. suis* isolates with complete genome sequences in our data set (Table S3). MGEs were identified using IslandViewer, using the most lenient criteria for identification to avoid underestimation of the length of these regions. The 29 isolates are from 9 different clusters, with 13 from cluster 1 (shown in red). We find that isolates with larger genomes have a greater combined MGE length (a), and that MGEs constitute a larger proportion of their overall genome size (b). Nevertheless, MGEs only explain a fraction of genome size variation (c). In addition, we find that MGEs tend to have lower average GC-contents than other genomic regions (c). This supports our hypothesis that within clusters the relationship between genome size and GC-content is largely explained by the variable presence of mobile genetic elements.

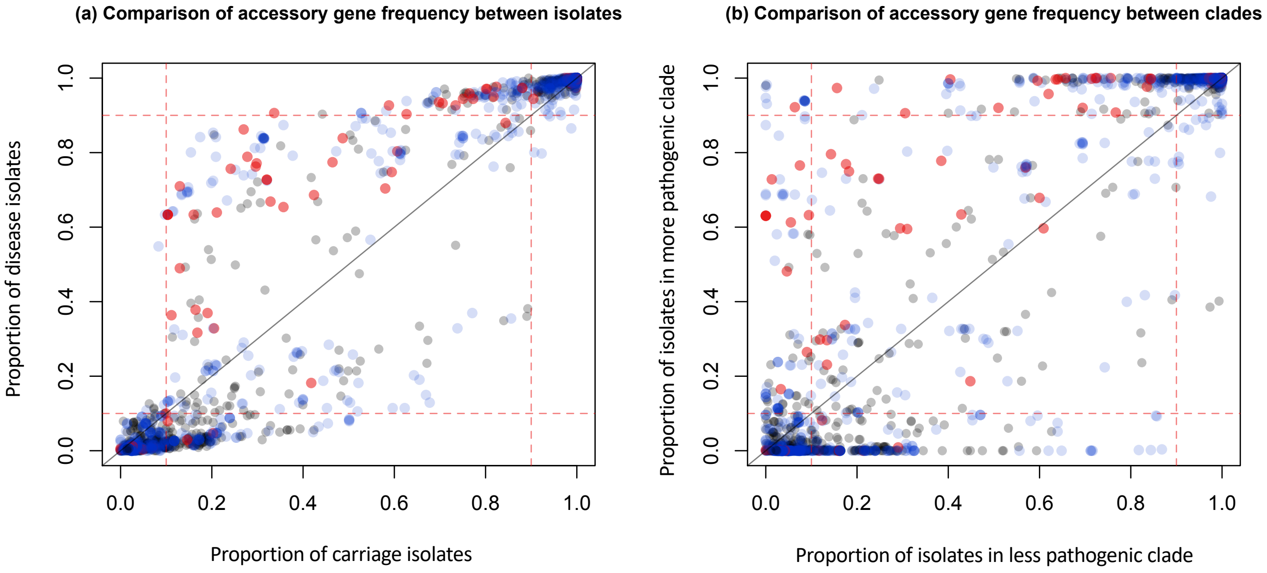

Fig. S9. Patterns of gene presence/absence in *S. suis*. Comparisons of the frequency of homologous genes across (a) all disease-associated isolates and all carriage isolates, and (b) across the more and less pathogenic clades. Solid lines indicate equal frequencies, and red dashed lines indicate frequencies of 10% and 90% in either category. Each point represents a homologous gene. Metabolism genes (identified through our COG classification) are shown in blue, virulence genes (identified in previous studies; see Figure S2) in red, and all other genes in grey. Gene frequencies are strongly correlated across carriage and disease isolates. No genes that are common in carriage isolates are absent from disease isolates (or vice versa). While gene frequencies are more divergent across the more and less pathogenic clades, no genes that are present in >90% of isolates in the less pathogenic clade are uniformly absent from isolates in the more pathogenic clade. This suggests that genome reduction is largely driven by the loss of broadly non-essential genes.

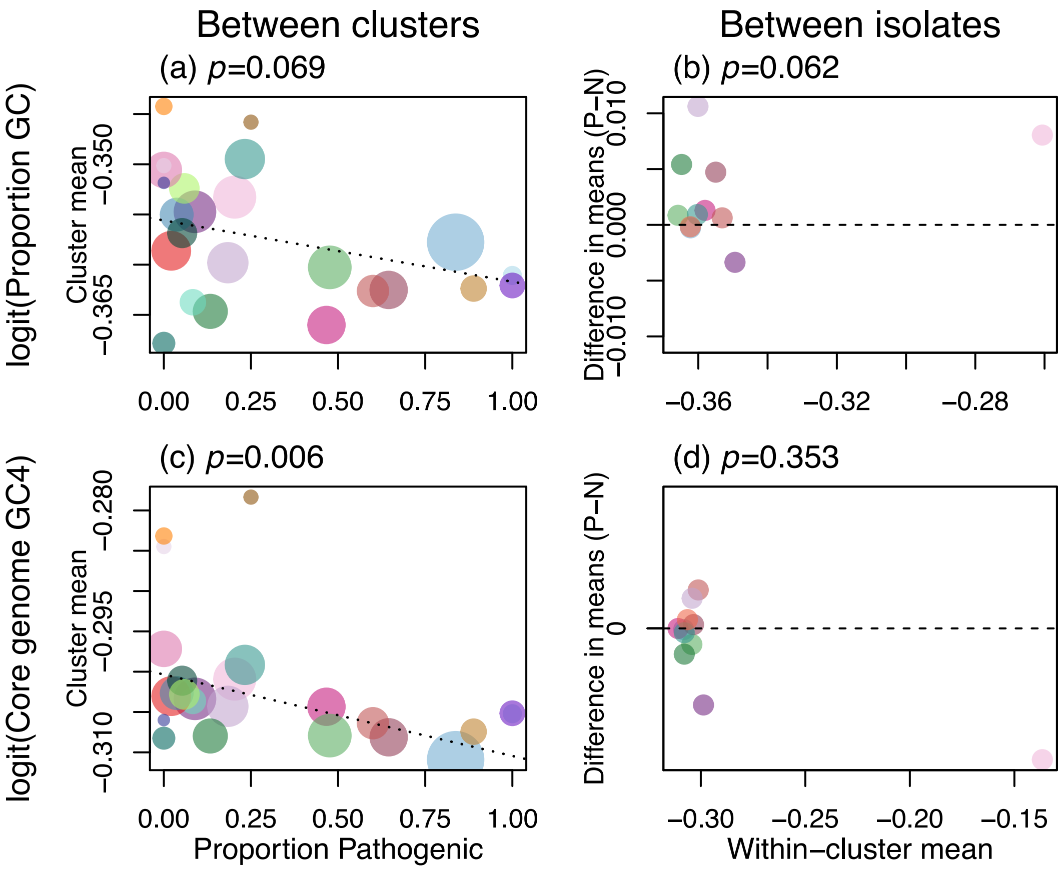

Fig. S10. GC content and pathogenicity *in S. suis*, removing the divergent clusters. Results are shown for *S. suis* after the removal of 10 divergent clusters with anomalous GC contents (see Figure S1). (a-b) overall genomic GC content, as in Figure 3h-i; (c-d) GC content at four-fold degenerate sites in protein-coding genes from the core genomes (i.e., that are present in all isolates). Core genome sites show the predicted association of low-GC with pathogenicity, but the effect is only evident over long periods of time (c). In the genome as a whole, the effect is masked by the absence of GC-poor mobile elements in smaller genomes.

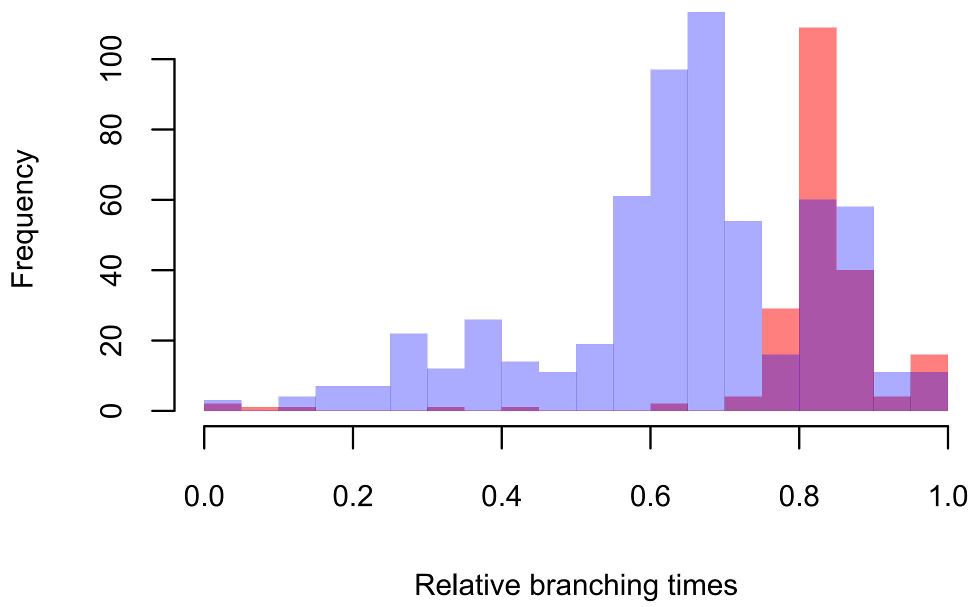

Fig. S11. **Pathogenicity and genealogy shape in *S. suis.*** The distribution of average branching times for our more (red) and less (blue) pathogenic clusters (as defined in Figure S1) as estimated by the *ltt* function in the *phytools* package in *R* (Revell 2012) applied to a midpoint-rooted neighbour-joining tree of our core gene alignment of non-clinical isolates. The root of each clade is zero on this timescale, with more recent branching times represented by larger values (bounded at 1). A non-parametric rank test shows significant difference between the two groups (Wilcoxon rank test, $p = 2.5 \times 10^{-8}$). This pattern suggests that more pathogenic clusters of *S. suis* have a lower effective population size and therefore a reduced efficacy of purifying selection. Disease isolates were omitted to avoid any bias driven by differences in sampling intensity between disease and carriage isolates, however this difference is robust to the inclusion of all isolates.

(a)

Matched pair	Cluster from more pathogenic clade	Cluster from less pathogenic clade
1	4	6
2	3	9
3	14	8
4	13	19
5	1	22
6	5	17
7	12	7
8	2	20
9	16	18

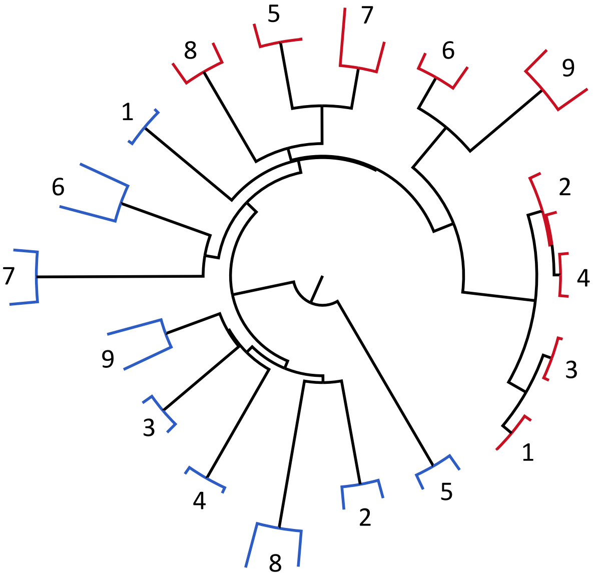

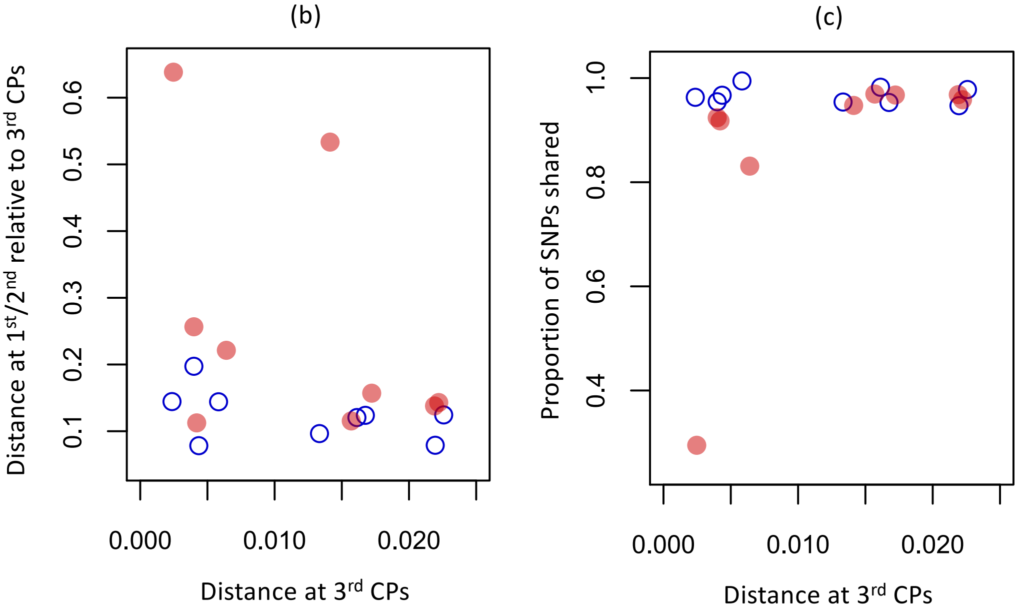

Fig. S12. Evidence of ineffective purifying selection and lower rates of recombination in more pathogenic clusters of *S. suis*. 9 pairs of isolates from 9 more pathogenic clusters were matched with 9 pairs of isolates from 9 less pathogenic clusters based on their pairwise distance at 3rd codon positions in an alignment of core genes. (a) Describes the clusters the pairs are from and their relative positions in a core genome neighbour-joining tree. These matched pairs were used to compare signatures of ineffective purifying selection (b) and recombination (c), across clusters from the more (red points) and less (blue circles) pathogenic clades. In 8/9 matched comparisons the cluster from the more pathogenic clade has the greater divergence at 1st and 2nd codon positions relative to divergence at 3rd codon positions (b). In the 4 matched comparisons with the lowest divergence at 3rd codon positions, the cluster from the more pathogenic clade has a lower proportion of SNPs where both variants are shared with isolates from other clusters (i.e. are more likely to have arisen through recombination) (c).

SI References

Fittipaldi, N., Segura, M., Grenier, D., and Gottschalk, M. (2012). Virulence factors involved in the pathogenesis of the infection caused by the swine pathogen and zoonotic agent *Streptococcus suis*. Future Microbiol. *7*, 259–279.

Holden, M.T.G., Hauser, H., Sanders, M., Ngo, T.H., Cherevach, I., Cronin, A., Goodhead, I., Mungall, K., Quail, M.A., Price, C., et al. (2009). Rapid evolution of virulence and drug resistance in the emerging zoonotic pathogen *Streptococcus suis*. PLoS One *4*, e6072.

Prüfer, T.L., Rohde, J., Verspohl, J., Rohde, M., de Greeff, A., Willenborg, J., and Valentin-Weigand, P. (2019). Molecular typing of *Streptococcus suis* strains isolated from diseased and healthy pigs between 1996-2016. PLoS One *14*, e0210801.

Revell, L.J. (2012). phytools: an R package for phylogenetic comparative biology (and other things). Methods in Ecology and Evolution *3*, 217–223.

Segura, M., Fittipaldi, N., Calzas, C., and Gottschalk, M. (2017). Critical *Streptococcus suis* Virulence Factors: Are They All Really Critical? Trends Microbiol. *25*, 585–599.

Wilson, T.L., Jeffers, J., Rapp-Gabrielson, V.J., Martin, S., Klein, L.K., Lowery, D.E., and Fuller, T.E. (2007). A novel signature-tagged mutagenesis system for *Streptococcus suis* serotype 2. Vet. Microbiol. *122*, 135–145.

Zheng, H., Luo, X., Segura, M., Sun, H., Ye, C., Gottschalk, M., and Xu, J. (2012). The role of toll-like receptors in the pathogenesis of *Streptococcus suis*. Veterinary Microbiology *156*, 147–156.
