# Supplementary material for: Genome Reduction Is Associated with Bacterial Pathogenicity across Different Scales of Temporal and Ecological Divergence
Source: Mol Biol Evol. 2020 Dec 12;38(4):1570–9. doi: 10.1093/molbev/msaa323 (PMC8042751; doi:10.1093/molbev/msaa323)
Supplement: msaa323_Supplementary_Data [file msaa323_Supplementary_Data.zip › Supplementary Tables-S4-S7.pdf]

Supplementary tables for

Genome reduction is associated with bacterial pathogenicity across different scales of temporal and ecological divergence

Gemma G. R. Murray, Jane Charlesworth, Eric L. Miller, Michael J. Casey, Catrin T. Lloyd, Marcelo Gottschalk, A. W. (Dan) Tucker, John J. Welch, and Lucy A. Weinert

Corresponding authors: Gemma G. R. Murray & Jane Charlesworth
Emails: ggrmurray@gmail.com; janepipistrelle@googlemail.com

This PDF file includes:

Tables S4 to S7

Other supplementary materials for this manuscript include the following:

Tables S1 to S3
Figures S1 to S12

Table S1 (separate file). Raw ancestral trait values for genome size and other traits for 31 phylogenetically-independent species pairs. For each genome trait there is a column for raw value in pathogen species, raw value in non-pathogen species and standard deviation of trait value.

Table S2 (separate file). Details of 1,318 genomes used in the between-species analysis.

Table S3 (separate file). Details of 1,079 *S. suis* genomes.

Table S4. Details of *S. suis* genetic clusters.

#	Genome size range (Mb)	# Isolate: Total (SP, RP, N)	Origin(s)	Dates
1	1.92 - 2.19	328 (199, 12, 41)	VN, UK, CA, CN, ES	2000 - 2016
2	2.09 - 2.29	45 (2, 12, 16)	UK, CA	2010 - 2016
3	2.04 - 2.26	78 (2, 18, 22)	UK, CA, US, CN	1988 - 2016
4	2.04 - 2.19	24 (4, 2, 4)	UK, ES	2010 - 2016
5	2.10 - 2.26	45 (11, 9, 11)	CA, UK, US, CN	1990 - 2016
6	1.96 - 2.30	61 (6, 3, 40)	UK, CN, CA	1987 - 2016
7	2.01 - 2.31	32 (2, 2, 26)	UK, CN, CA	2008 - 2016
8	1.98 - 2.36	73 (8, 4, 47)	UK, CA, CN, US, ES	1983 - 2016
9	1.99 - 2.54	70 (2, 3, 51)	UK, CA, CN, US	1989 - 2016
10	2.21 - 2.55	55 (3, 7, 33)	UK, CA, CN, US	1988 - 2016
11	2.04 - 2.29	15 (1, 1, 7)	UK, CN, CA	2009 - 2014
12	2.07 - 2.36	9 (0, 0, 9)	CN, CA	2013 - 2016
13	2.05 - 2.30	13 (7, 1, 1)	UK, CA	2010 - 2013
14	2.05 - 2.30	12 (4, 4, 0)	CA, UK	1989 - 2016
15	2.01 - 2.02	3 (0, 0, 2)	CA	1991 - 2016
16	2.27 - 2.42	6 (1, 3, 0)	CA, UK	1989 - 2014
17	2.13 - 2.26	13 (0, 1, 11)	UK, CA	1994 - 2016
18	2.04 - 2.21	52 (0, 1, 46)	CN, UK, CA	2009 - 2013
19	2.14 - 2.21	19 (0, 1, 18)	CN	2013
20	2.10 - 2.45	28 (0, 1, 26)	CN, CA, UK	1989 - 2016
21	2.28 - 2.53	37 (0, 0, 37)	CN	2013 - 2014
22	2.30 - 2.48	20 (1, 0, 16)	CN, UK, ES, CA	2011 - 2016
23	1.97 - 2.24	7 (1, 0, 1)	UK, CA	2008 - 2014
24	2.23 - 2.39	4 (0, 0, 3)	UK, CA	2013 - 2014
25	2.24 - 2.39	5 (1, 0, 4)	UK, CA	2010 - 2016
26	2.29 - 2.46	5 (0, 0, 3)	CA, US	2016
27	2.15 - 2.40	4 (0, 0, 4)	UK, CN	2013
28	2.09 - 2.26	4 (0, 0, 4)	CN, UK	2013 - 2014
29	2.07 - 2.17	4 (0, 0, 4)	CN, CA	2013 - 2016
30	2.17 - 2.36	4 (1, 0, 3)	CA, UK, CN	1988 - 2013
31	2.18	1 (0, 0, 0)	UK	2010
32	2.46	1 (0, 0, 1)	CN	2013
33	2.15	1 (0, 0, 1)	UK	2013
34	2.16	1 (0, 0, 1)	CN	2013

Note: SP: Systemic pathogen, RP: Respiratory pathogen, C: Carriage; VN: Vietnam, UK: United Kingdom, CA: Canada, CN: China, ES: Spain.

Table S5. Robustness of between-species analyses.

Trait	Outlier(s) removed p [n]	"changed ecology" removed p ($n = 19$)	"changed ecology" only p ($n = 12$)
Genome size, $\log_{10}(bp)$	0.0307* [30]	0.0180*	0.4526
Total genome size (incl. plasmids) $\log_{10}(bp)$	0.0321* [30]	0.0264*	0.3774
Coding genome size, $\log_{10}(bp)$	0.0102* [30]	0.0210*	0.1300
#CDS, $\log_{10}(number)$	0.0414* [30]	0.0480*	0.2790
#Metabolic genes, $\log_{10}(number)$	0.0148* [30]	0.0212*	0.8091
logit(proportion metabolic)	0.0979 [30]	0.9855	0.7803
Non-coding genome size, $\log_{10}(bp)$	0.2312 [29]	0.7728	0.5670
logit(proportion functional)	0.3431 [29]	0.3095	0.2008
logit(proportion GC)	0.0404* [30]	0.3087	0.0487*

Note: Permutation tests of the difference in trait values between pathogenic and non-pathogenic sister species, calculated using standardised independent contrasts. The first column shows results after the removal of outlying points (identified visually). The second columns show subsets of the data, separating the $n = 12$ points with a changed ecology, defined as pairs where the pathogen species was scored as facultatively intracelluar and the non-pathogen as extracellular, and/or the pathogen as host-restricted, and the non-pathogen as not host restricted. * $p < 0.05$.

Table S6. Robustness of the association between genome size and pathogenicity in between-cluster data.

Dataset: #clusters (# genomes):	A: 33 (1078)	B: 24 (883)	C: 31 (1060)	D: 14 (948)
	$\hat{\beta}$ (p)	$\hat{\beta}$ (p)	$\hat{\beta}$ (p)	$\hat{\beta}$ (p) [$\hat{\lambda}$]; GLS regression
Genome size, $\log_{10}(bp)$	-0.031 (0.0054)	-0.042 (0.0022)	-0.042 (0.0008)	-0.032 (0.0000) [1.16]
Coding genome size, $\log_{10}(bp)$	-0.025 (0.0067)	-0.034 (0.0020)	-0.034 (0.0013)	-0.043 (0.0086) [1.14]
#CDS, $\log_{10}(number)$	-0.024 (0.0341)	-0.038 (0.0060)	-0.035 (0.0044)	-0.049 (0.0034) [1.15]

Note: A: Dataset contains the 33/34 genetic clusters containing isolates that could be unambiguously assigned as “clinical” or “non-clinical”; B: Dataset retains only clusters for which at least 2/3 of isolates could be unambiguously assigned; C: Measures of genome size were calculated exclusively from isolates assigned as “non-clinical” (which were found in 31/34 clusters); D: Dataset contains only “large” clusters, which contained at least 20 isolates. All entries report results from linear regression of mean trait value onto the proportion of clinical strains in each cluster, using sample-size-weighted least squares (datasets A-C), or generalized-least squares, correcting for covariation due to shared ancestry (dataset D). $\hat{\beta}$: best-fit regression slope; p: the associated p-value; $\hat{\lambda}$: the best-fit value of Pagel’s λ for phylogenetically-corrected regression.

Table S7. Individual COG categories for metabolic genes.

	COG Category	logit(*proportion*) (A) p ($\bar{\delta x}$)	(B) p ($\hat{\beta}$)	(C) $p(\bar{\delta})$	$\log_{10}(number)$ (A) p ($\bar{\delta x}$)	(B) p ($\hat{\beta}$)	(C) $p(\bar{\delta})$
C	Energy production and conversion	0.193 (0.034)	0.015 (-0.035)	0.012 (0.008)	0.703 (-0.027)	0.009 (-0.040)	0.065 (0.005)
E	Amino acid transport and metabolism	0.745 (0.012)	0.008 (-0.029)	0.158 (0.003)	0.534 (-0.046)	0.006 (-0.034)	0.805 (-0.001)
F	Nucleotide transport and metabolism	0.042 (0.070)	0.143 (0.014)	0.075 (0.003)	0.974 (0.011)	0.247 (0.009)	0.947 (0.000)
G	Carbohydrate transport and metabolism	0.300 (-0.040)	0.000 (0.046)	0.201 (-0.004)	0.065 (-0.094)	0.019 (0.041)	0.020 (-0.007)
H	Coenyme transport and metabolism	0.190 (0.032)	0.000 (0.045)	0.461 (0.002)	0.746 (-0.028)	0.000 (0.040)	0.403 (-0.001)
I	Lipid transport and metabolism	0.713 (-0.011)	0.292 (0.010)	0.449 (0.001)	0.381 (-0.068)	0.614 (0.005)	0.343 (-0.002)
P	Inorganic ion transport and metabolism	0.328 (0.022)	0.130 (-0.018)	0.835 (0.000)	0.615 (-0.038)	0.135 (-0.023)	0.395 (-0.003)
Q	Secondary metabolites biosynthesis, transport and catabolism	0.369 (-0.038)	0.533 (0.008)	0.595 (0.004)	0.262 (-0.094)	0.841 (0.003)	0.935 (0.001)

Note: All analyses match those shown in Figure 3a-c, and Figure S3m-o, but with a single class of metabolic gene. A: The between-species data; p: permutation P-value, $\bar{\delta x}$: mean different in independent contrasts P-N; B: The between-cluster *S. suis* data; p: regression P-value, $\hat{\beta}$: best-fit regression slope; C: the between-isolate *S. suis* data; p: permutation P-value, $\bar{\delta}$: difference in within-cluster means, P-N. Negative values of the test statistics ($\bar{\delta x}$, $\hat{\beta}$ or $\bar{\delta}$) indicate a tendency for pathogens to preferentially lose metabolic genes.
